# Supplementary material for: Candidate genes involved in biosynthesis and degradation of the main extracellular matrix polysaccharides of brown algae and their probable evolutionary history
Source: BMC Genomics. 2024 Oct 10;25:950. doi: 10.1186/s12864-024-10811-3 (PMC11468063; doi:10.1186/s12864-024-10811-3)
Supplement: Supplementary file 1 — Supplementary Material 1 [file 12864_2024_10811_MOESM1_ESM.pdf]

Candidate genes involved in biosynthesis and degradation of the main extracellular matrix polysaccharides of brown algae and their probable evolutionary history

Lisa Mazéas[†,1], Ahlem Bouguerba-Collin[†,1], J. Mark Cock[1], France Denoeud[2], Olivier Godfroy[1], Loraine Brillet-Guéguen[1,3], Tristan Barbeyron[1], Agnieszka P. Lipinska[4], Ludovic Delage[1], Erwan Corre[3], Elodie Drula[5,6,7], Bernard Henrissat[5,6,8], Mirjam Czjzek[1], Nicolas Terrapon[5,6], Cécile Hervé[1,*]

[1]Sorbonne Université, CNRS, Integrative Biology of Marine Models Laboratory, Station Biologique de Roscoff, Roscoff, France

[2]Génomique Métabolique, Genoscope, Institut François Jacob, CEA, CNRS, Université Evry, Université Paris-Saclay, Evry, 91057, France

[3]CNRS, Sorbonne Université, FR2424, ABiMS-IFB, Station Biologique, Roscoff, France

[4]Department of Algal Development and Evolution, Max Planck Institute for Biology, 72076, Tübingen, Germany.

[5]Aix Marseille Univ, CNRS, UMR 7257 AFMB, Marseille, France

[6]INRAE, USC 1408 AFMB, Marseille, France

[7]INRAE, Aix-Marseille Univ., UMR1163 BBF, Marseille, France

[8]Department of Biotechnology and Biomedicine, Technical University of Denmark, Kgs. Lyngby, Denmark

Supplementary Figure legends

Supplementary Fig. S1. Phylogenetic trees of key enzymes involved in the synthesis of GDP-mannose, a precursor for the synthesis of both alginates and fucans.

A) The bifunctional mannose-6-phosphate isomerases (MPI)/mannose-1-phosphate guyanylyltransferases (MPG) and **B)** the bifunctional phosphomannomutases (PMM)/ phosphoglucomutase (PGM).

Supplementary Fig. S2. Clustering and phylogenetic position of brown algal proteins within the GDP-mannose/UDP-glucose dehydrogenases (GMD/UGDs) superfamily.

A) Heatmap representing BLASTP+ Score Ratios (BSRs) for protein sequence pairwise alignments. The colour code correlates BSRs. Two main groups of Stramenopile sequences are clearly apparent: one containing the GMD sequences and one harbouring the UGD sequences. The bacterial sequences from *Actinobacteria* and *Pseudomonadota* are distantly related to these two groups. **B)** Uncompressed version of the phylogenetic tree of GMDs shown in Fig. 2A of the main manuscript.

Supplementary Fig. S3. Protein alignment of GMD sequences.

Selected GMD sequences from brown algae were aligned against the bacterial GMD sequence from *Pseudomonas aeruginosa* (pdbcode: 1mv8). All these sequences have been biochemically characterized. The secondary structure assignments shown above the sequences are those determined for 1mv8. The red arrowheads and stars below the alignment indicate the conserved catalytic residues involved in the NAD(H) fixation and the mannuronic acid, respectively.

Supplementary Fig. S4. Uncompressed version of the phylogenetic tree of ManC5-E proteins shown as an inset in Fig. 2B of the main manuscript

Supplementary Fig. S5. Protein alignment of ManC5-E sequences.

Selected ManC5-E sequences from brown algae were aligned against the bacterial ManC5-E sequence from *Pseudomonas syringae* (pdbcode: 4NK6). The secondary structure assignments shown above the sequences are those determined for 4NK6. The red arrowheads below the alignment indicate the conserved catalytic residues, notably from the typical DPHD motif.

Supplementary Fig. S6. Uncompressed version of the phylogenetic tree of PL41 proteins shown in Fig. 3A of the main manuscript

Supplementary Fig. S7. Protein alignment of PL41 sequences.
The SBBH10631.1 sequence from *Saccharina japonica* has been biochemically characterized.

Supplementary Fig. S8. Phylogenetic tree of DEHU reductases, known to be active in alginate degradation in brown algae

Supplementary Fig. S9. Protein alignment of DEHU reductase sequences.
Selected DEHU-reductase sequences from brown algae were aligned against a mouse succinic semialdehyde reductase sequence (pdbcode: 2c91) from the aldo-keto reductase (AKR) superfamily. The secondary structure assignments shown above the sequences are those determined for 2c91. The red arrowheads below the alignment indicate key catalytic residues for 2c91.

Supplementary Fig. S10. Uncompressed versions of the phylogenetic tree of GH88 proteins shown in Fig. 3B of the main manuscript

Supplementary Fig. S11. Protein alignment of GH88 sequences.
Selected GH88 sequences from brown algae were aligned against the bacterial GH88 sequence from *Bacillus* sp. (pdbcode: 1vd5), an unsaturated glucuronyl hydrolase involved in the degradation of glycosaminoglycan. The secondary structure assignments shown above the sequences are those determined for 1vd5. The red arrowheads below the alignment indicate the likely candidate catalytic residues for glycosyl hydrolysis in 1vd5.

Supplementary Fig. S12. Phylogenetic trees of key enzymes involved in the synthesis of GDP-fucose
Enzymes involved in the synthesis of GDP-fucose through the *de novo* pathway: **A)** GDP-mannose 4,6-dehydratases (GM46D) and **B)** the GDP-L-fucose synthetases (GFS); and through the salvage pathway: **C)** fucokinase (FK) and **D)** GDP-fucose pyrophosphorylase (GFPP).

Supplementary Fig. S13. Uncompressed version of the phylogenetic tree of GT10 proteins shown in Fig. 5A of the main manuscript

Supplementary Fig. S14. Protein alignment of GT10 sequences.

Full version of the alignment shown in Fig. 5B of the main manuscript. The secondary structure assignments shown above the sequences are those determined for 2nzw. The red and green arrowheads below the alignment indicate the conserved catalytic residues involved in the fixation of GDP-fucose and in the fixation of the acceptor in 2nzw, respectively.

Supplementary Fig. S15. Uncompressed version of the phylogenetic tree of GT41 proteins shown in Fig. 6 of the main manuscript

Supplementary Fig. S16. Protein alignment of GT41 sequences.

Selected GT41 sequences from brown algae were aligned against a human GT41 sequence with a GlcNAc characterized activity (top sequence; pdbcode: 3pe3) and a GT41 sequence from *Arabidopsis thaliana* with a fucosyltransferase characterized activity (bottom sequence, pdbcode: 7y4i). The secondary structure assignments shown above and below the sequences are those determined for 3pe3 and 7y4i, respectively. The arrowheads below the alignment indicate the candidate catalytic residues for the UDP fixation by 3pe3 in red, and for the GDP fixation by 7y4i in green.

Supplementary Fig. S17. Overlay of a human GT41 crystallised structure with protein models of brown algal GT41s.

The characterized human GT41 (pdb code: 3pe3), bound with UDP-GlcNAc, was superimposed with AlphaFold2 models of GT41 proteins from: A, B) *Ectocarpus* sp.7 (accession number: Ec-01_007580.1) and C, D) *Fucus distichus* (accession number: contig3697_1). Only low similarity can be detected, mostly concentrated in the donor sites (A, C). The conservation of these residues important for UDP-sugar binding could indicate that these enzymes also use UDP-activated sugars as donors. None of the Alphafold2 models of the two algal GT41s analysed showed similarity to the GDP-binding pocket of the plant GT41 (*Arabidopsis thaliana,* pdb code: 7y4i; data not shown). Larger views of the overall structures are indicated (B, D). In addition, among characterized GT41 multiple specificities have been reported (activities with 3 different sugar-donors have been described so far), which is also reflected by the large divergence of the phylogenetic tree. It is thus not possible, at this stage, to predict any substrate specificities for the different phylogenetic clades of GT41.

Supplementary Fig. S18. Uncompressed version of the phylogenetic tree of GT74 proteins shown in Fig. 7 of the main manuscript

Supplementary Fig. S19. Protein alignment of GT74 sequences.

Selected GT74 sequences from brown algae and Alveolata were aligned against the biochemically characterized GT74 from the amoeba *Dictyostelium discoideum*. These proteins being bimodular, the alignment has been made on the GT74 domain only.

Supplementary Fig. S20. Uncompressed version of the phylogenetic tree of GT23 proteins shown in Fig. 8B of the main manuscript

Supplementary Fig. S21. Protein alignment of GT23 sequences.

Full version of the alignment shown in Fig. 8C of the main manuscript. The secondary structure assignments shown above the sequences are those determined for the human FUT8 (pdbcode: 6X5R). The red arrowheads below the alignment indicate the conserved catalytic residues involved in the fixation of GDP-fucose in 6X5R.

Supplementary Fig. S22. Uncompressed version of the phylogenetic tree of ST proteins shown in Fig. 9A of the main manuscript.

Supplementary Fig. S23. Protein alignment of STs from group 10.

Selected sequences of brown algal STs (orthologues from group 10) were aligned against 2 echinoderm ST sequences taken from Fig. 10B of the main manuscript. The red and brown stars below the alignment locate the PAPS fixation sites with respectively the 5′-phosphosulfate and 3′-phosphoadenosine binding sites.

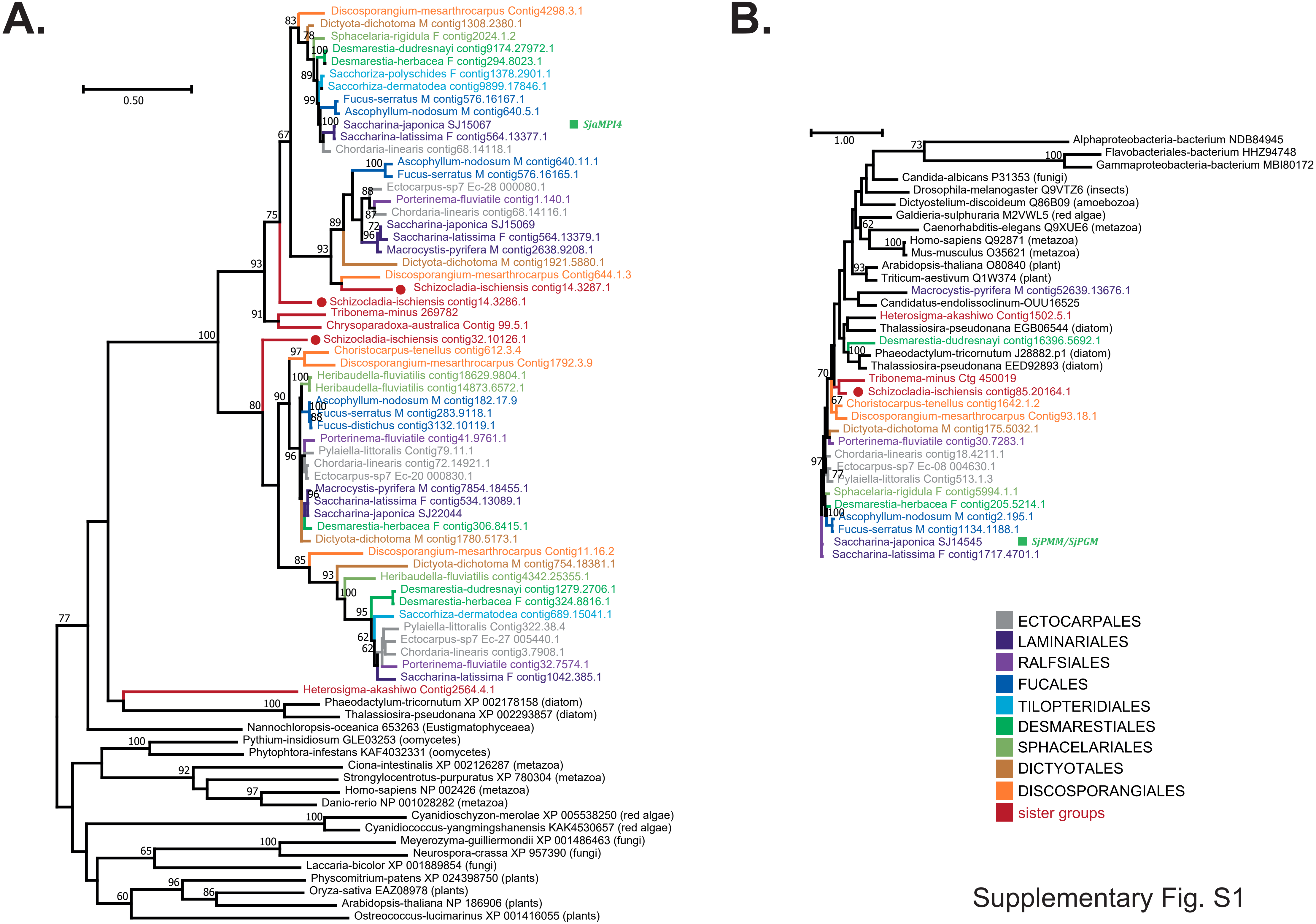

Supplementary Fig. S1

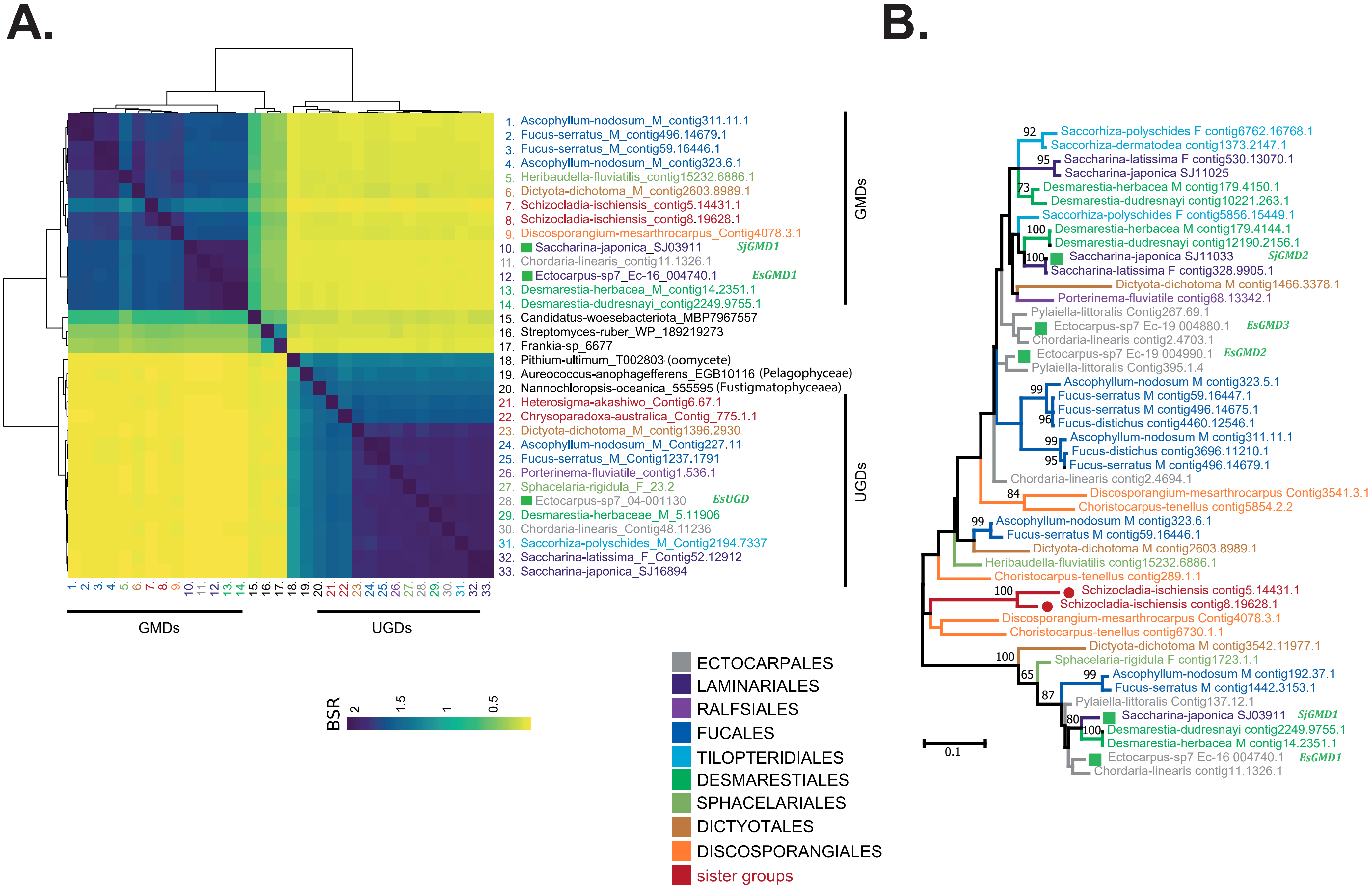

Supplementary Fig. S2

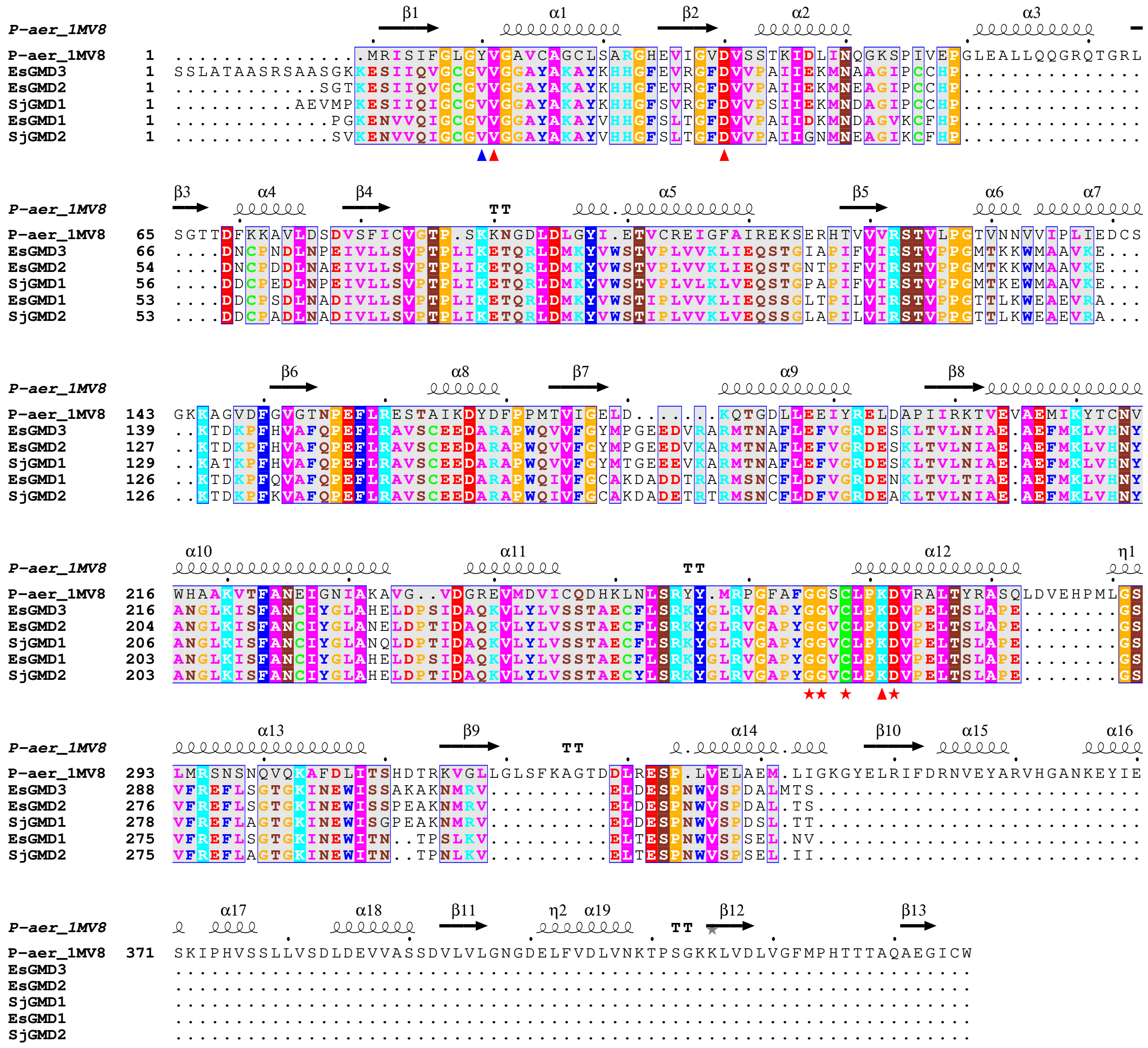

Supplementary Fig. S3

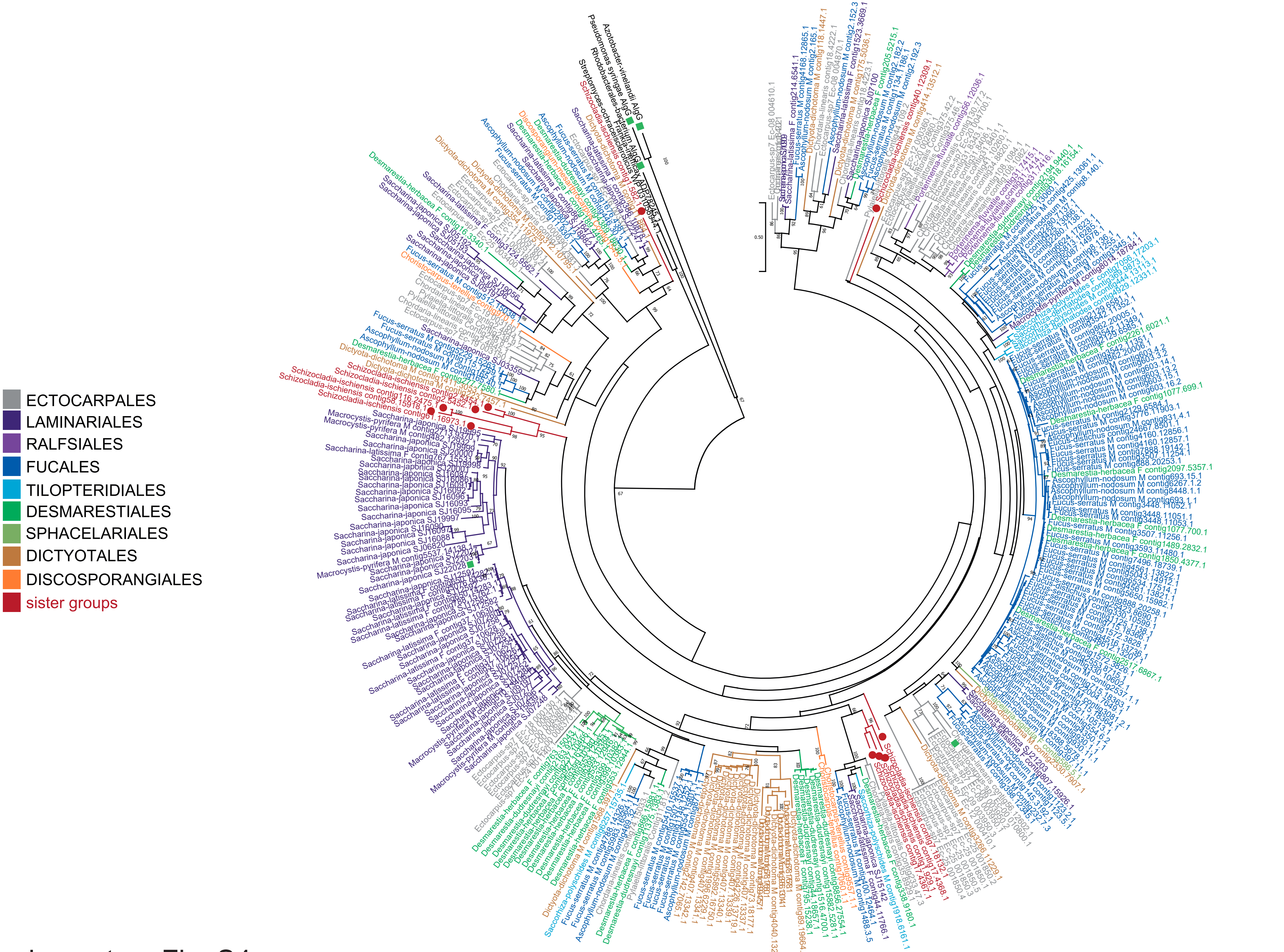

Supplementary Fig. S4

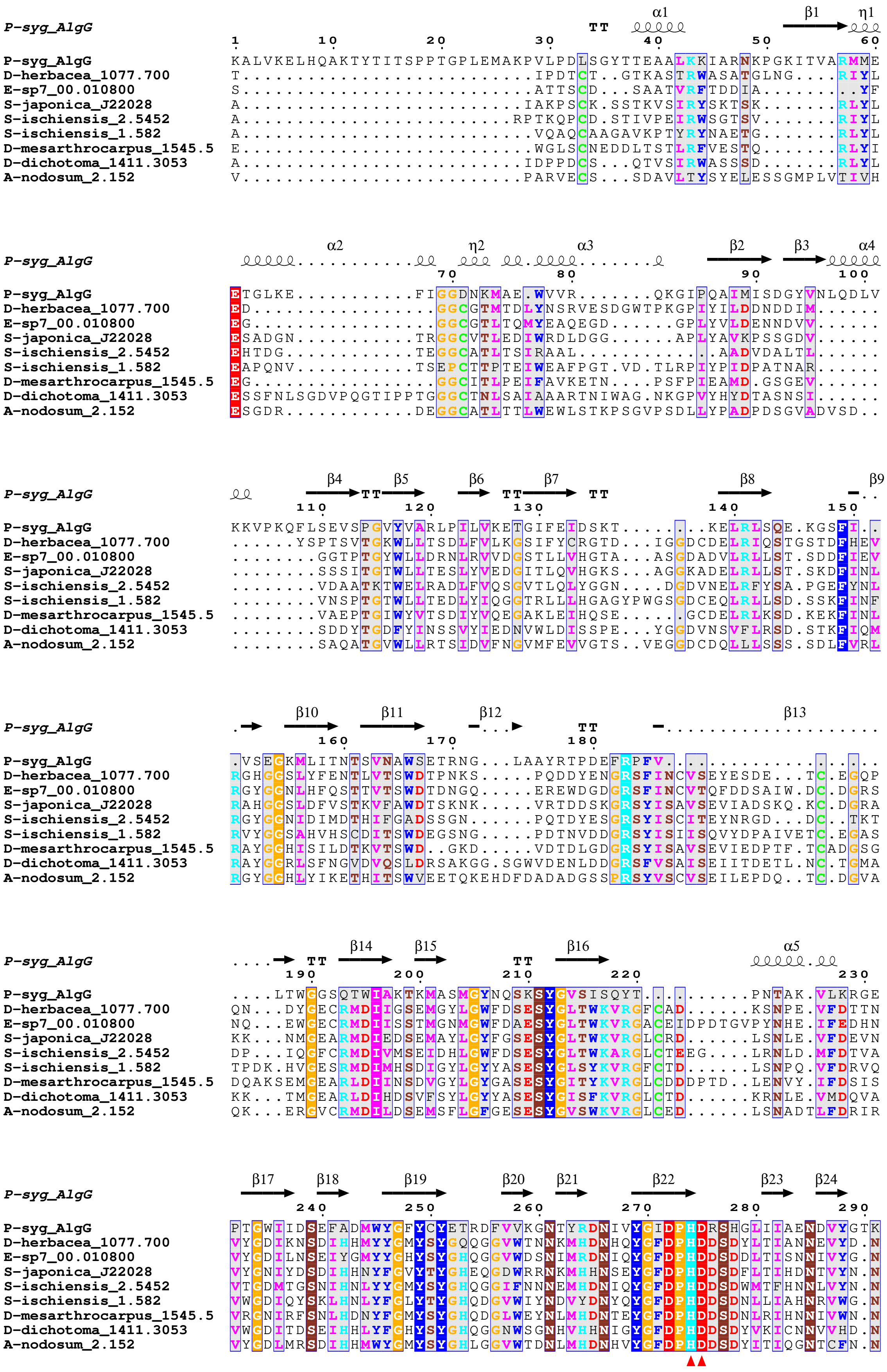
P-syg_AlgG
TT α1 β1 η1
1 10 20 30 40 50 60
P-syg_AlgG KALVKELHQAKTYTITSPPTGPLEMAKPVLPDLSGYTTEAALKKIARNKPGKITVARMME
D-herbacea_1077.700 T...........................IPDTCT..GTKASTRWASATGLNG....RIYL
E-sp7_00.010800 S...........................ATTSCD...SAATVRFTDDIA.........YF
S-japonica_J22028 A...........................IAKPSCK.SSTKVSIRYSKTSK.......RLYL
S-ischiensis_2.5452 A..........................RPTKQPCD.STIVPEIRWSGTSV.......RIYL
S-ischiensis_1.582 A...........................VQAQCAAGAVKPTYRYNAETG.......RLYL
D-mesarthrocarpus_1545.5 E...........................WGLSCNEDDLTSTLRFVESTQ.......RLYI
D-dichotoma_1411.3053 A...........................IDPPDCS...QTVSIRWASSSD.......RLYL
A-nodosum_2.152 V...........................PARVECS...SDAVLTYSYELESSGMPLVTIVH
P-syg_AlgG
α2 η2 α3 β2 β3 α4
70 80 90 100
P-syg_AlgG ETGLKE..........FIGGDNKMAE.WVVR.......QKGIPQAIMISDGYVNLQDLV
D-herbacea_1077.700 ED..............GGCGTMTDLYNSRVESDGWTPKGPIYILDDNDDIM......
E-sp7_00.010800 EG..............GGCGTLTQMYEAQEGD......GPLYVLDENNDVV......
S-japonica_J22028 ESADGN..........TRGGCVTLEDIWRDLDGG......APLYAVKPSSGDV......
S-ischiensis_2.5452 EHTDG...........TEGGCATLTSIRAAL..........AADVDALTL......
S-ischiensis_1.582 EAPQNV..........TSEPCTTPTEIWEAFPGT.VD.TLRPIYPIDPATNAR......
D-mesarthrocarpus_1545.5 EG..............GGCITLPEIFAVKETN....PSFPIEAMD.GSGEV......
D-dichotoma_1411.3053 ESSFNLSGDVPQGTIPPTGGGCTNLSAIAARTNIWAG.NKGPVYHYDTASNSI......
A-nodosum_2.152 ESGDR...........DEGGCATLTTLWEWLSTKPSGVPSDLLYPADPDSGVADVSD..
P-syg_AlgG
β4 TT β5 β6 TT β7 TT β8 β9
110 120 130 140 150
P-syg_AlgG KKVPKQFLSEVSPGVYVARLPILVKETGIFEIDSKT.......KELRLSQE.KGSFI..
D-herbacea_1077.700 ......YSPTSVTGKWLLTSDLFVLKGSIFYCRGTD...IGGDCDELRIQSTGSTDFHEV
E-sp7_00.010800 ........GGTPTGYWLLDRNLRVVDGSTLLVHGTA...ASGDADVLRLLST.SDDFIEV
S-japonica_J22028 ........SSSITGTWLLTESLYVEDGITLQVHGKS...AGGKADELRLLST.SKDFINL
S-ischiensis_2.5452 ........VDAATKTWELRADLFVQSGVTLQLYGGN....DGDVNELRFYSA.PGEFYNL
S-ischiensis_1.582 ........VNSPTGTWLLTEDLYIQGGTRLLLHGAGYPWGSGDCEQLRLLSD.SSKFINF
D-mesarthrocarpus_1545.5 ........VAEPTGIWYVTSDIYVQEGAKLEIHQSE.....GCDELRLKSD.KEKFINL
D-dichotoma_1411.3053 ........SDDYTGDFYINSSVYIEDNVWLDISSPE..YGGDVNSVFLRSD.STKFIQM
A-nodosum_2.152 ........SAQATGVWLLRTSIDVFNGVMFEVVGTS..VEGGDCDQLLLSSS.SDLFVRL
P-syg_AlgG
β10 β11 β12 TT β13
160 170 180
P-syg_AlgG .VSEGKMLITNTSVNAWSETRNG...LAAYRTPDEFRPFV..................
D-herbacea_1077.700 RGHGGSLYFENTLVTSWDTPNKS.....PQDDYENGRSFINCVSEYESDE...TC.EGQP
E-sp7_00.010800 RGYGGNLHFQSTTVTSWDTDNGQ.....EREWDGDGRSFINCVTQFDDSAIW.DC.DGRS
S-japonica_J22028 RAHGGSLDFVSTKVFAWDTSKNK.....VRTDDSKGRSYISAVSEVIADSKQ.KC.DGRA
S-ischiensis_2.5452 RGYGGNIDIMDTHIFGADSSGN......PQTDYESGRSYISCITEYNRGD...DC..TKT
S-ischiensis_1.582 RVYGGSAHVHSCDITSWDEGSNG.....PDTNVDDGRSYISIISQVYDPAIVETC.EGAS
D-mesarthrocarpus_1545.5 RAYGGHISILDTKVTSWD..GKD.....VDTDLGDGRSYISAVSEVIEDPTF.TCADGSG
D-dichotoma_1411.3053 RAYGGRLSFNGVDVQSLDRSAKGG.SGWVDENLDDGRSFVSAISEIITDETL.NC.TGMA
A-nodosum_2.152 RGYGGHLYIKETHITSWVEETQKEHDFDADADGSSPRSYVSCVSEILEPDQ..TC.DGVA
P-syg_AlgG
TT β14 β15 TT β16 α5
190 200 210 220 230
P-syg_AlgG ....LTWGGSQTWIAKTKMASMGYNQSKSYGVSISQYT.........PNTAK.VLKRGE
D-herbacea_1077.700 QN...DYGECRMDIIGSEMGYLGWFDSESYGLTWKVRGFCAD......KSNPE.VFDTTN
E-sp7_00.010800 NQ...EWGECRMDIISSTMGNMGWFDAESYGLTWKVRGACEIDPDTGVPYNHE.IFEDHN
S-japonica_J22028 KK...NMGEARMDIEDSEMAYLGYFGSESYGLTWKVRGLCRD......LSNLE.VFDEVN
S-ischiensis_2.5452 DP...IQGFCRMDIVMSEIDHLGWFDSESYGLTWKARGLCTEEG....LRNLD.MFDTVA
S-ischiensis_1.582 TPDK.HVGESRMDIMHSDIGYLGYYASESYGLSYKVRGFCTD......LSNPQ.VFDRVQ
D-mesarthrocarpus_1545.5 DQAKSEMGEARLDIINSDVGYLGYGASESYGITYKVRGLCDDDPTD..LENVY.IFDSIS
D-dichotoma_1411.3053 KK...TMGEARLDIHDSVFSYLGYYASESYGISFKVRGLCTD......KRNLE.VMDQVA
A-nodosum_2.152 QK...ERGVCRMDILDSEMSFLGFGESESYGVSWKVRGLCED......LSNADTLFDRIR
P-syg_AlgG
β17 β18 β19 β20 β21 β22 β23 β24
240 250 260 270 280 290
P-syg_AlgG PTGWIIDSEFADMWYGFYCYETRDFVVKGNTYRDNIVYGIDPHDRSHGLIIAENDVYGTK
D-herbacea_1077.700 VYGDIKNSDIHMYYGMYSYGQQGGVWTNNKMHDNHQYGFDPHDDSDYLTIANNEVYD.N
E-sp7_00.010800 VYGDILNSEIYGMYYGHYSYGHQGGVWDSNIMRDNIQYGFDPHDDSDDLTISNNIVYG.N
S-japonica_J22028 VYGNIYDSDIHHNYFGVYTYGHEQGDWRRNKMHHNSEYGFDPHDDSDFLTIHDNTVYN.N
S-ischiensis_2.5452 VTGDMTGSNIHNLYYGMYSYGHQGGIFNNNEMHDNIQYGFDPHDDSDWMTFHNNLVYN.N
S-ischiensis_1.582 VWGDIQYSKLHNLYFGLYTYGHQDGVWIYNDVYDNYQYGFDPHDDSDNLLIAHNRVWG.N
D-mesarthrocarpus_1545.5 VRGNIRFSNLHDNYFGMYSYGHQDGLWEYNLMHDNTEYGFDPHDDSDNLRIHNNIVWN.N
D-dichotoma_1411.3053 VWGDITDSEIHHLYFGHYSYGHQGGNWSGNHVHHNIGYGFDPHDDSDYVKICNNVVHD.N
A-nodosum_2.152 VYGDLMRSDIHHMWYGMYSYGHLGGVWTDNLMHDNHVYGFDPHDDSDYITIQGNTCFN.N

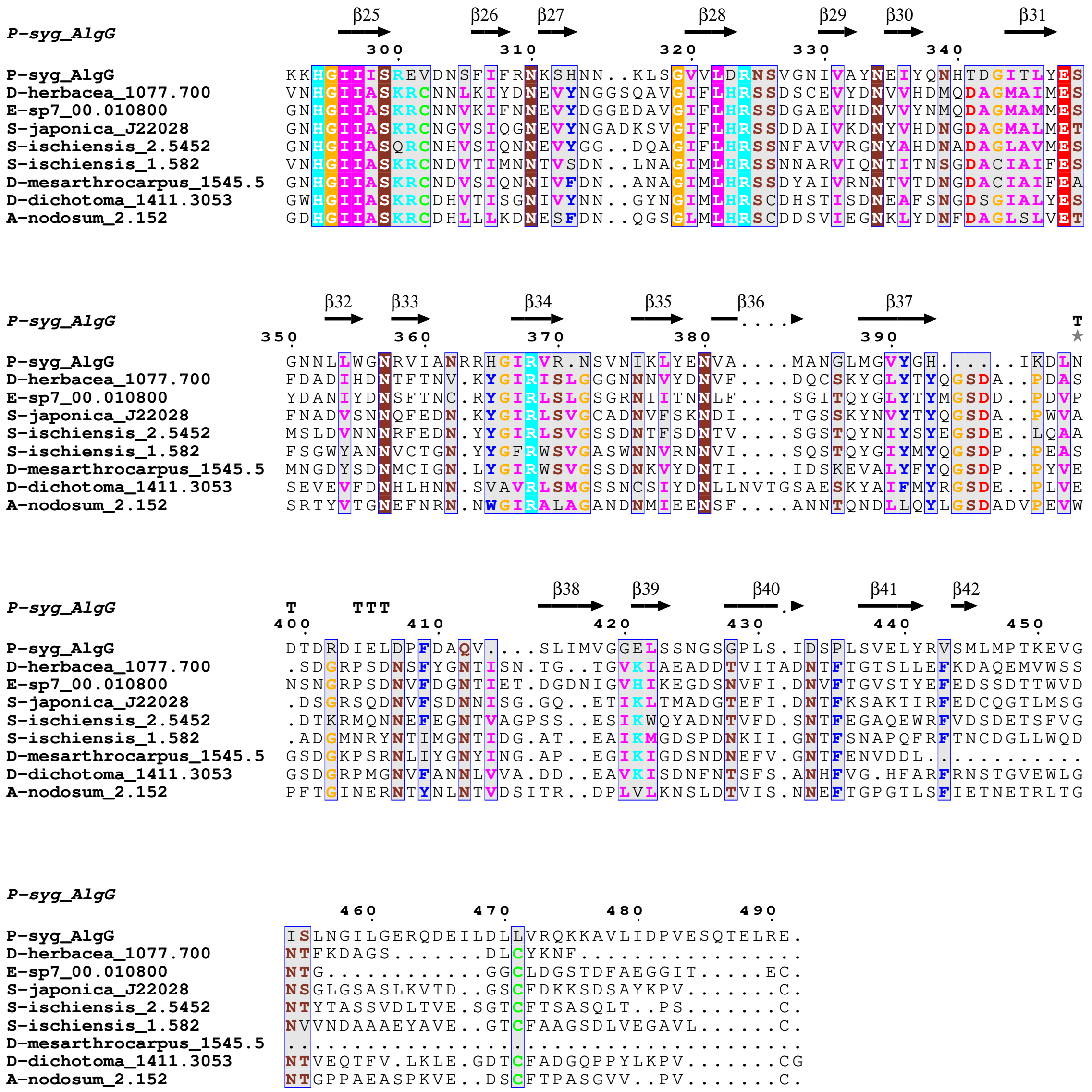

Supplementary Fig. S5

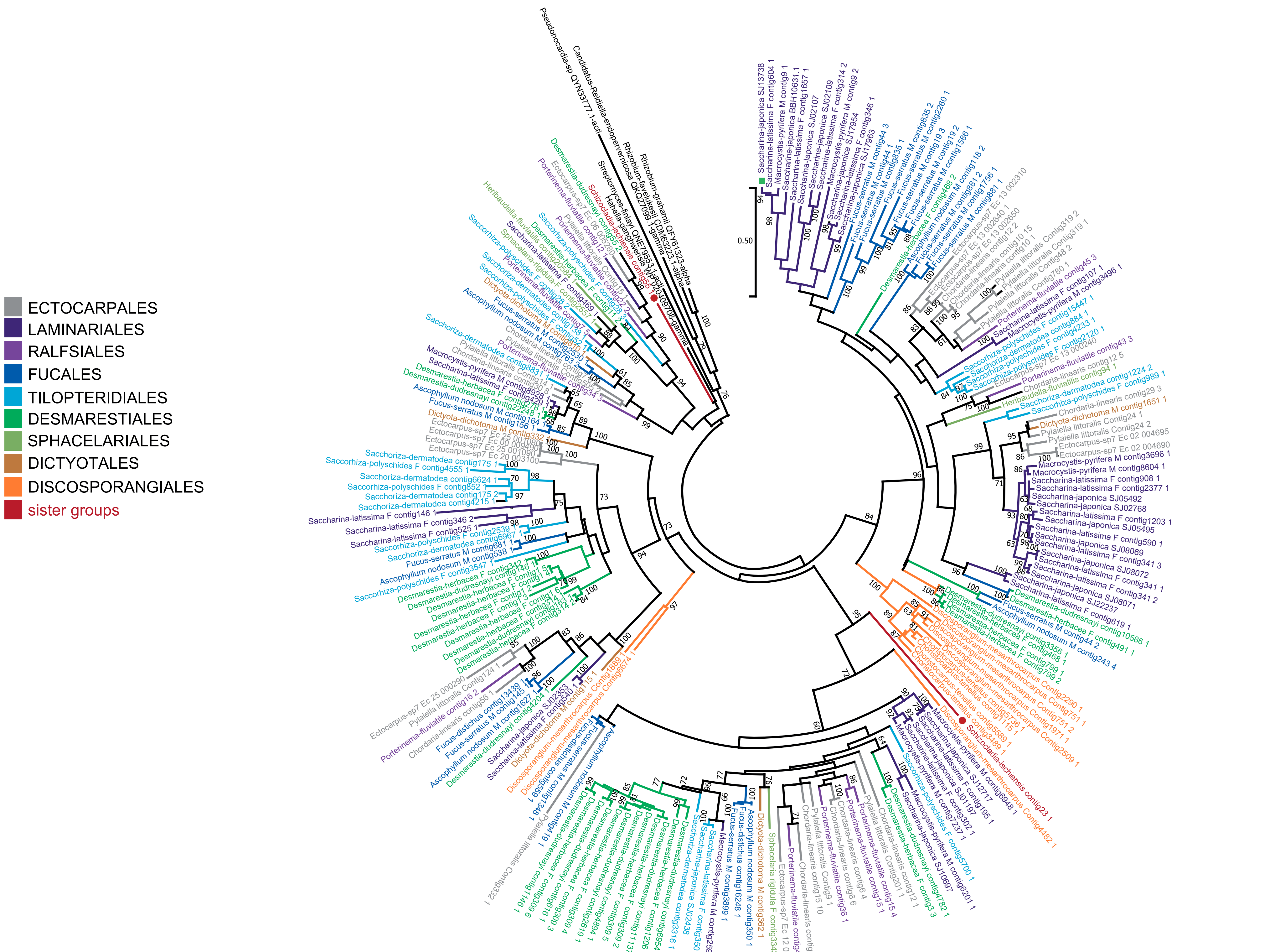

Supplementary Fig.S6

```
                            1        10
S-japonica_BBH10631.1       V...VSG..TCL.......DLL.........................LP..............................
D-dichotoma_362_1           ...............................................................................
E-sp7_25.000290             P..............................................................................
F-serratus_145_1            ...............................................................................
S-ischiensis_23_1           ...............................................................................
E-sp7_02.004695             VDMIFSSIPTCIYRCAHFAHLLIYYFSPPEPNRDRAHHQVLLAETTTQPTNIGHGNMAVTANLRRQQQQPYQETAKPVS
S-ischiensis_55_1           ...............................................................................
H-ganghwensis_WP020409708   N..................KLW.........................................................

                                               20                       30        40          50        60
S-japonica_BBH10631.1       .............AIIMLATLTPAVTAA..............TTYKVTPNGKWTSSSG...YSSSSKIYSLPGALAIAK
D-dichotoma_362_1           ...R.......TAALGLLISITYGVLG...............EEFKLEAGADFEAE.................CLMKVG
E-sp7_25.000290             ....ARGGAFFGFAIVVLVALVTWGII...............ALVEVAEDNNSSSDSGD.FADSRDVDSDLQALLDDAS
F-serratus_145_1            ....VKGGAFFGFAIVVAAALLTWGIIA..............IVNEATKGSGGSSDGGDEYYTPPDGLSEIQDALNDAS
S-ischiensis_23_1           ...............................................................................
E-sp7_02.004695             KNSPVTGAAAATAARLATAALALTAASA..............ATYEVSPDGD...............PMTLTAALEVAG
S-ischiensis_55_1           ..........................................ATYPVSGGG..................SINAAISQAQ
H-ganghwensis_WP020409708   .............TVLLFVSVFTQTVWAMPVQNSYEEFDYPNFTTFYVSPTGDDNNSGSE....QAPLQSINMALSLAQ

                                      70        80        90            100                110       120
S-japonica_BBH10631.1       AGDTILLADGTYTDRLESYRDGKSSSPIKIKGG.....EGAKIK..AGSPS......VHIRHSWILLQGFTVDGKHESS
D-dichotoma_362_1           PGDSCYLEKGDYEHDGLTRTHGTEDDRIVIRGH.....PEACIKG.SNTQD....RVFQVAHNYYTIEDICFDGKHGS.
E-sp7_25.000290             PGDTIYIPAGTHDVSLTTVVDGTEDEPITIRNELGTSAEDCVLR...GDDR...ARVLEVLHNHYIIQGFTIDGDDGSG
F-serratus_145_1            PGDIIEIPAGTYYIDLVTVVDGTEDEPITLRGAAGTSRADVVLKG.NGDS.....RVLDIRHSWYIIEDFTINGEVDEG
S-ischiensis_23_1           .....RVAAGHYTDNFFTSMNGEKGCIIKIACE.....NGAVLHP.TGEKD....RMVEQMHSYHHWDNCEFDGQ....
E-sp7_02.004695             AGDTISLGDGIYREPLVTMKAGVEGNPLVIEGG....RDAVVNYYTGDKSIMWSQKVVDIRHSWVTLRGFTIDGQIDDA
S-ischiensis_55_1           PGDVVSVASGTYREDLRTVRDGESGRPITIRCA.....EGAVLHG.AGDTV....AIFHVEHSYITVEGCTLDGHFCNE
H-ganghwensis_WP020409708   AGDRVYLQPGHYYENVRSVRHGQADMPIIIHGS.....KSAVLH...GAQG....RILEINHSYIHLRNFTIDGQVGSG

                            130      140                   150       160       170       180       190
S-japonica_BBH10631.1       SKKSSYVNKCVSVEGTGK............SSSDPLKGFVMKDMVIKNCGTECVRLKNFVTNAEIKDNTITKCGVYDFK
D-dichotoma_362_1           ....DYVATAIYVLGEDK...KSDLKVNGIESKSSVTGLIMRNLEIKNFGSECIHFRYFVTWTEMEGCTVQHCGIDAFE
E-sp7_25.000290             ....DYKEQLLYIEGSRD...PSEVD....GVDSSITGVNVQGMVLKNAGKECLRIRYFATNCVVQVNEIGPCGIQDFQ
F-serratus_145_1            ...EEYREQLIFVEGSSD...PEETD....DGDVSIEGIVIQNMRLRNAGKECLRFKYWVVNSVIQSNDIRYCGVEDFQ
S-ischiensis_23_1           ....GKAATLMMILGIDR...KKKISSSDGDIDSSVTGVRITDCHFHHATAECMHLRYFTTYTEVTGCDFHDCGMKNFG
E-sp7_02.004695             DHEDSFVDKCIFVEGQAA...PEDVEYDGRTVETALIGTVIDSMKIQNCGMECIRMRNFVTNTVIIDNDIEDCGIYDYR
S-ischiensis_55_1           NVDSCYRDKLMYVQRDRDAAVRAQEISYKGRRFRSSINGLVLRRNRFLNGGECLRLRYFVTYSVITQNEFRNCGVHDFE
H-ganghwensis_WP020409708   NTMEDFRDILVYVKGNEP..............QQGPTGFVMRAMSLKNAGGECVRLRYYVTEAEIFDNTFYNCGVYDFK

                                200       210                 220       230       240       250
S-japonica_BBH10631.1       FD.EGGDNGEGIYIGTSSTQWKNGE...................DRCNDNRISGNTISTYGSECVDVKEGCSGTTIEEN
D-dichotoma_362_1           NG.GGGKVGEAVYIGTALDQVDDDKAPEVKLRSGSAD...NVDSDVCSYNWVHHNTFRTYGNECVDVKEGSTMNLIENN
E-sp7_25.000290             QD.SDGSNGEGVYIGTALNQIDEKN........GDD......VVDECTGIVVMNNEIDTQGSEGVDIKEGSYGNVVYGN
F-serratus_145_1            LG.SGGSNGEGVYIGTASGQWNDDR..................EDVCRGIVVQYNYIDTQGSEGVDIKEGSIENLVQYN
S-ischiensis_23_1           EGCDDGKVGECIYIGTSISQWDDGKHPH..PERGTDQPDKPDYDPDRCQFNHIHNNMKSNGNECSEVKENSRYNLIEYN
E-sp7_02.004695             YT.FDGKVGEAIYIGTSSNQWADGADGR..VTHG.........PDGCNYNLVTGNKLVPRGNECVDIKEGATMNVVEYN
S-ischiensis_55_1           FN.HGGKNGEGIYCGTSSEQWGDGKNPE....NG.........PDQSNYNEITDNTFVTNGNECVELKEGAEFNTVAGN
H-ganghwensis_WP020409708   FN.NGGKNGEAIYVGTSSNQWDDGKNPT....GG.........PDPSNHNWIHHNKFLTNGNECVDIKEGAEYNLVEYN

                                260       270       280       290       300       310                320
S-japonica_BBH10631.1       KCSDQRQALTGCFSVRGDDNTVRYNTAKDCEGVGVRLGGAKVG.KHQYGKDNDVYENTITDAEMG..........FMRI
D-dichotoma_362_1           VCEQQRDPNSGCFGLRGSENTVRYNEIAECYGAGVRIGG.....DKHFGDGNHIYGNVIKNTGNG..........AFNV
E-sp7_25.000290             TIRGQMDEETGGISLRGDENIVVDNHVIDGSGTCIRVGGFEED.GVQYGEDNEVYENTLDGCLNS..........AVKV
F-serratus_145_1            DITGEIDGEGGGIGLRGDYNIVRYNFVFDTLGACVRIGGNEDDNDRQWGVNNEVYENDLDGCIAG..........AIKV
S-ischiensis_23_1           TCSHQMDPNSGGFSCRGEHNTFRYNEVRDCVGAGIRLGSDD...NKNYGHSNNVYGNKFEGNGQGNGGCGQGKGRVGVYN
E-sp7_02.004695             DCEDQRDVESGCYDSRGNENTFRYNTAENCLGGGIRLGGHEID.GITYGVDNHVYGNSFRETGDG..........SIKA
S-ischiensis_55_1           DCSGQRDPESAGMGCRSDRNTFRGNTIRGSVGAGIRIGGHNAG.GKTWGENNNICNNQLVSNDYA..........GLKL
H-ganghwensis_WP020409708   ICSGQQDPNSAGFDSRGDHNIFRYNVSHDNKGAGVRLGGNTVD.GRQYGVNNQVYGNLLYNNAQA..........GVKL

                                 330        340       350
S-japonica_BBH10631.1       TAMPQGRICENKCK.GGSCDITADIGVRDIRE....TWDED.......C...............................
D-dichotoma_362_1           MKPDQGAVCENSIS..GATSVSAEGAYKD.......EPDFELSASTGDCDSYPGSLDGSNDSAPPESAPTD.........
E-sp7_25.000290             NESPQ.LICDNTVI..LAADPTDDELYAVVRGDAAEEFTDTVSA...ACTGDKTVFFLPDHY..................
F-serratus_145_1            NDTPQ.ILCDNDVE.....APSDEDNWVAIIGDAADEFESITD....SCPT.LMSFALPDQAVPGEVGGSEDFGDDDVDD
S-ischiensis_23_1           TDNQEQNICGNEGNDDGACDAEYDAGWGGIEG.GGDEDDED..................................QDEED
E-sp7_02.004695             RRDPQGVICENSCE.DGDCDVSEEGDDEEIED....SWDQE.......CPSSLPSVPFIDDLGTP...............
S-ischiensis_55_1           MTGPQAQVC.......................................................................
H-ganghwensis_WP020409708   MAAPQEKVCGNPTS.........DNTEGDIMGSSAMSYDPELS.....C...............................

S-japonica_BBH10631.1       ......
D-dichotoma_362_1           ......
E-sp7_25.000290             ......
F-serratus_145_1            HSDSVS
S-ischiensis_23_1           R.....
E-sp7_02.004695             ...TLT
S-ischiensis_55_1           ......
H-ganghwensis_WP020409708   ......
```

Supplementary Fig. S7

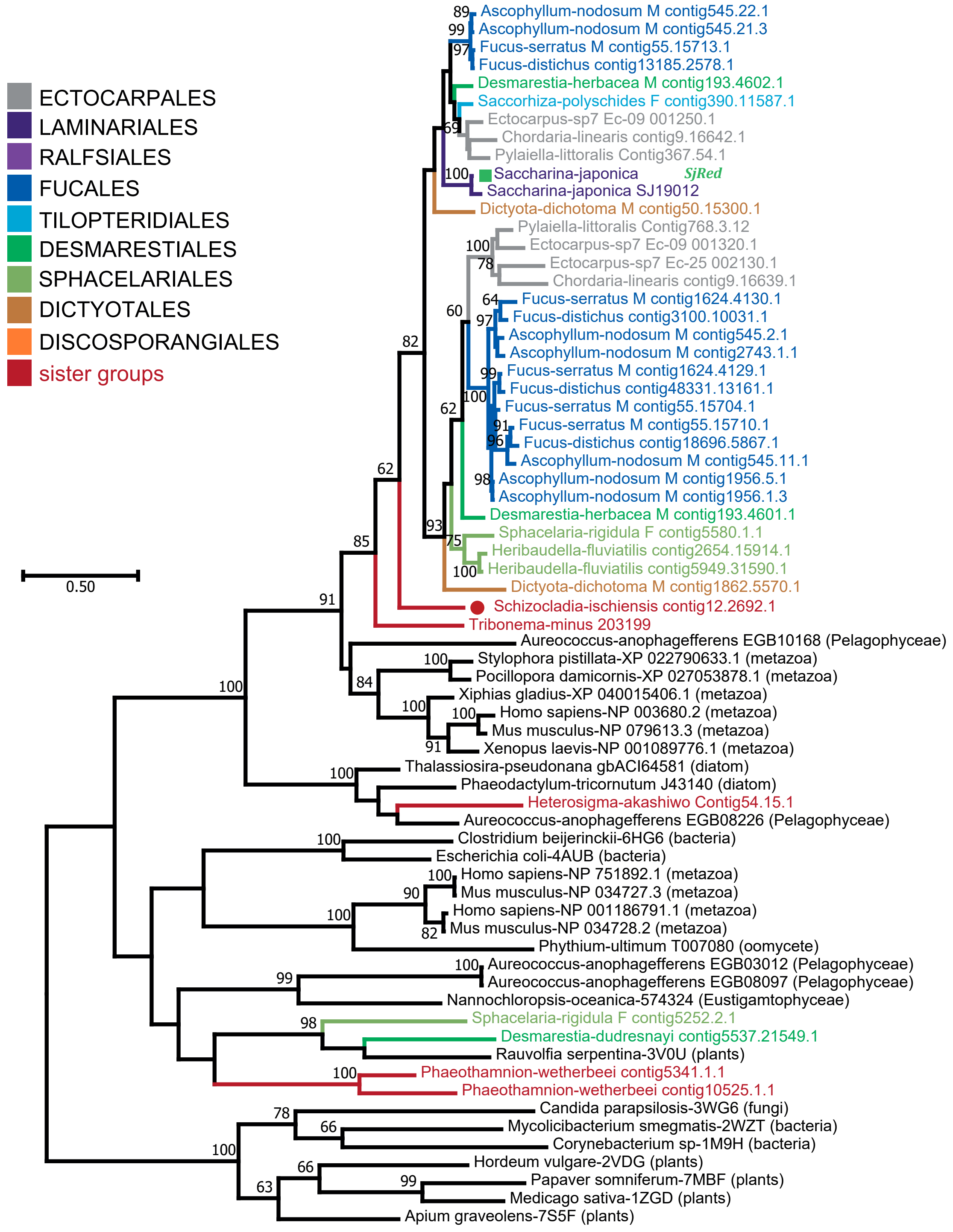

Supplementary Fig. S8

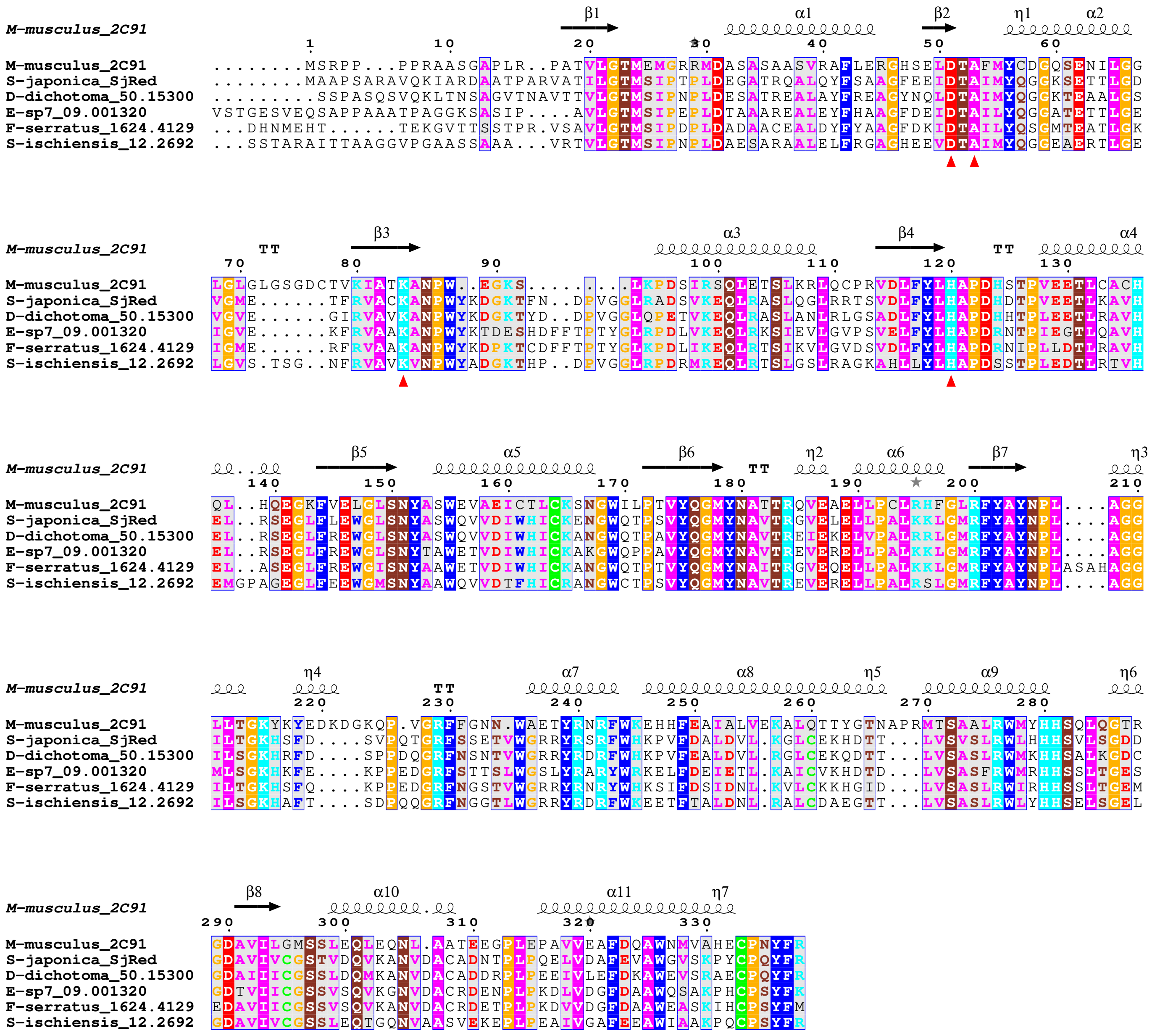

Supplementary Fig. S9

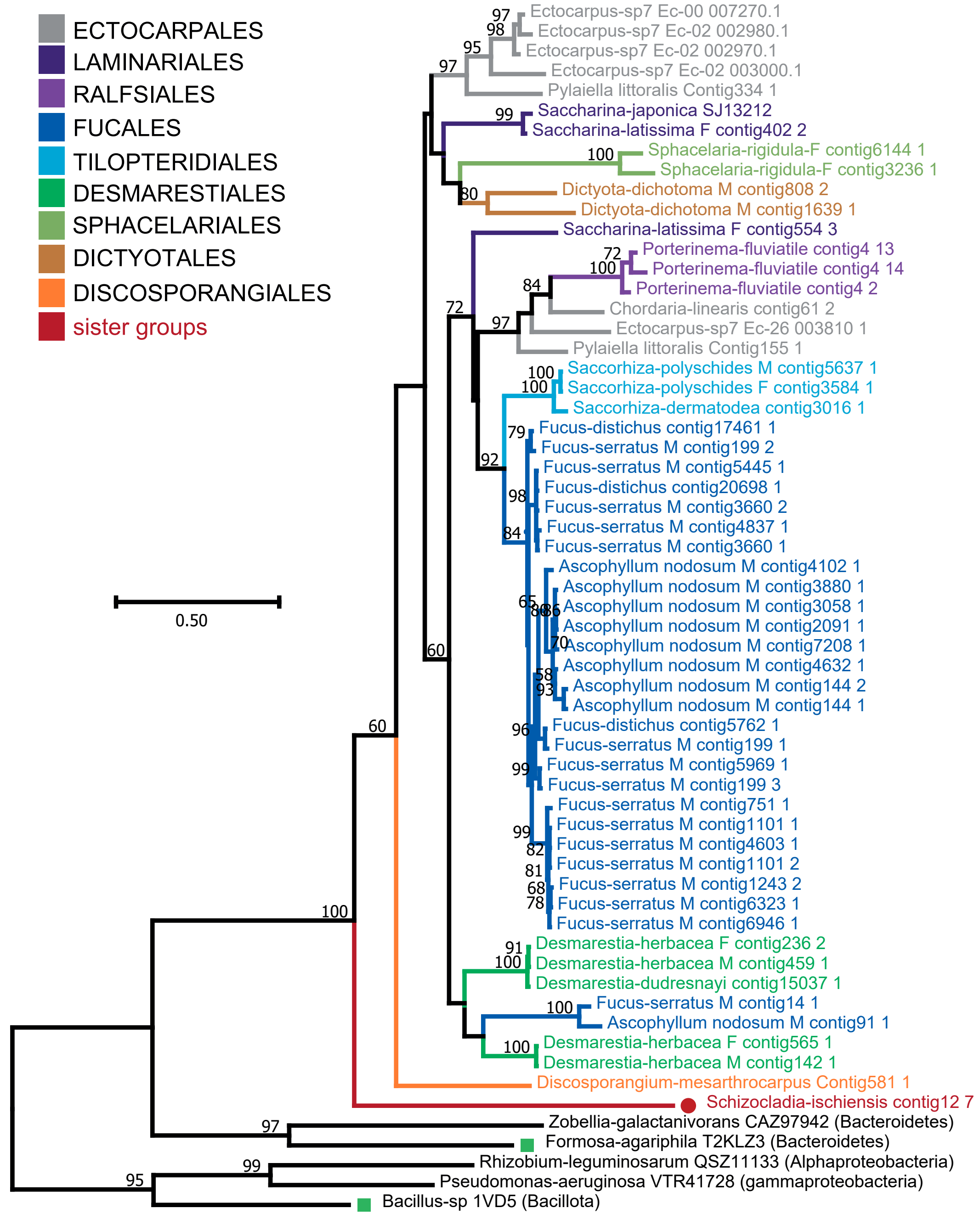

Supplementary Fig. S10

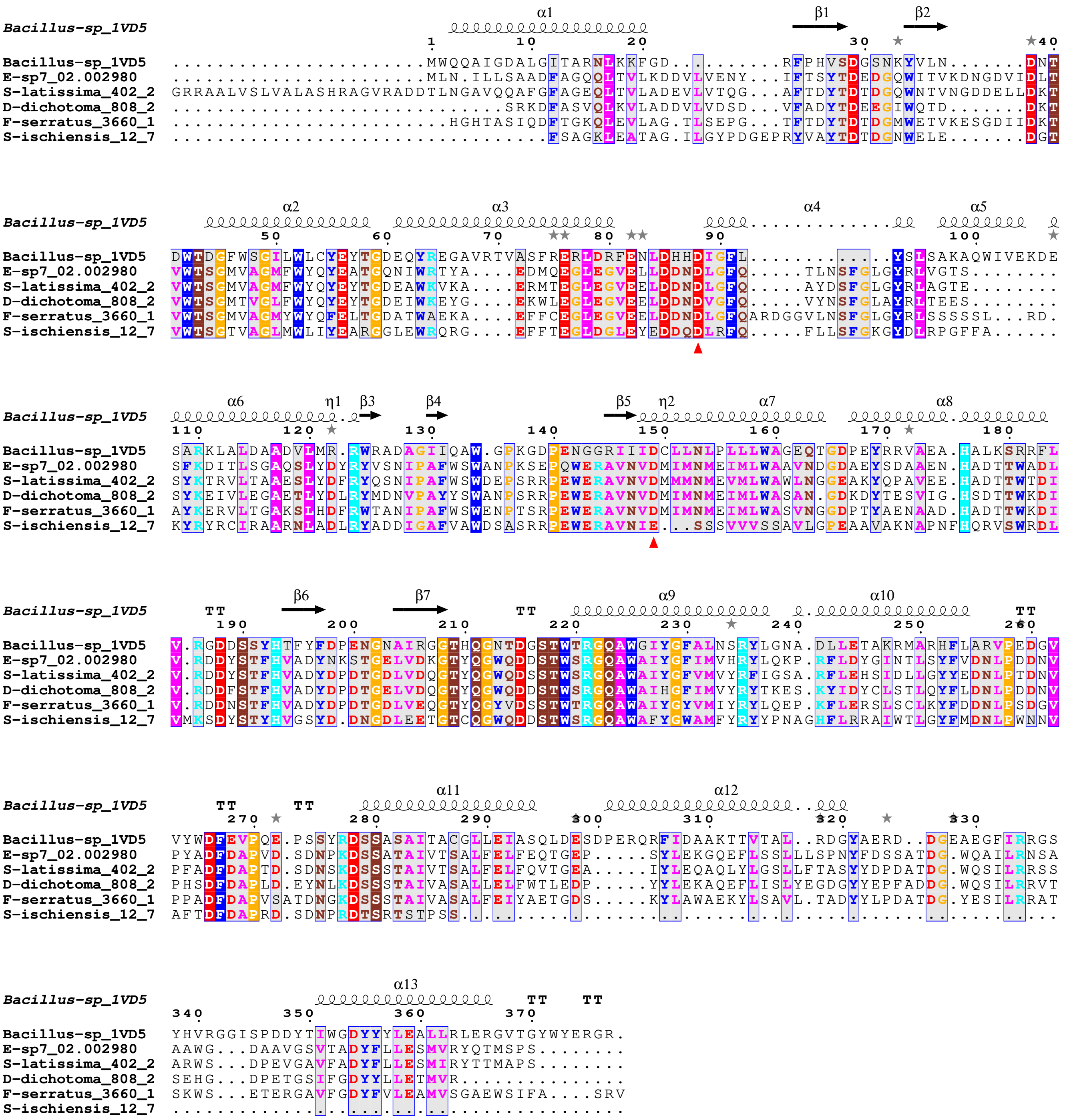

Supplementary Fig. S11

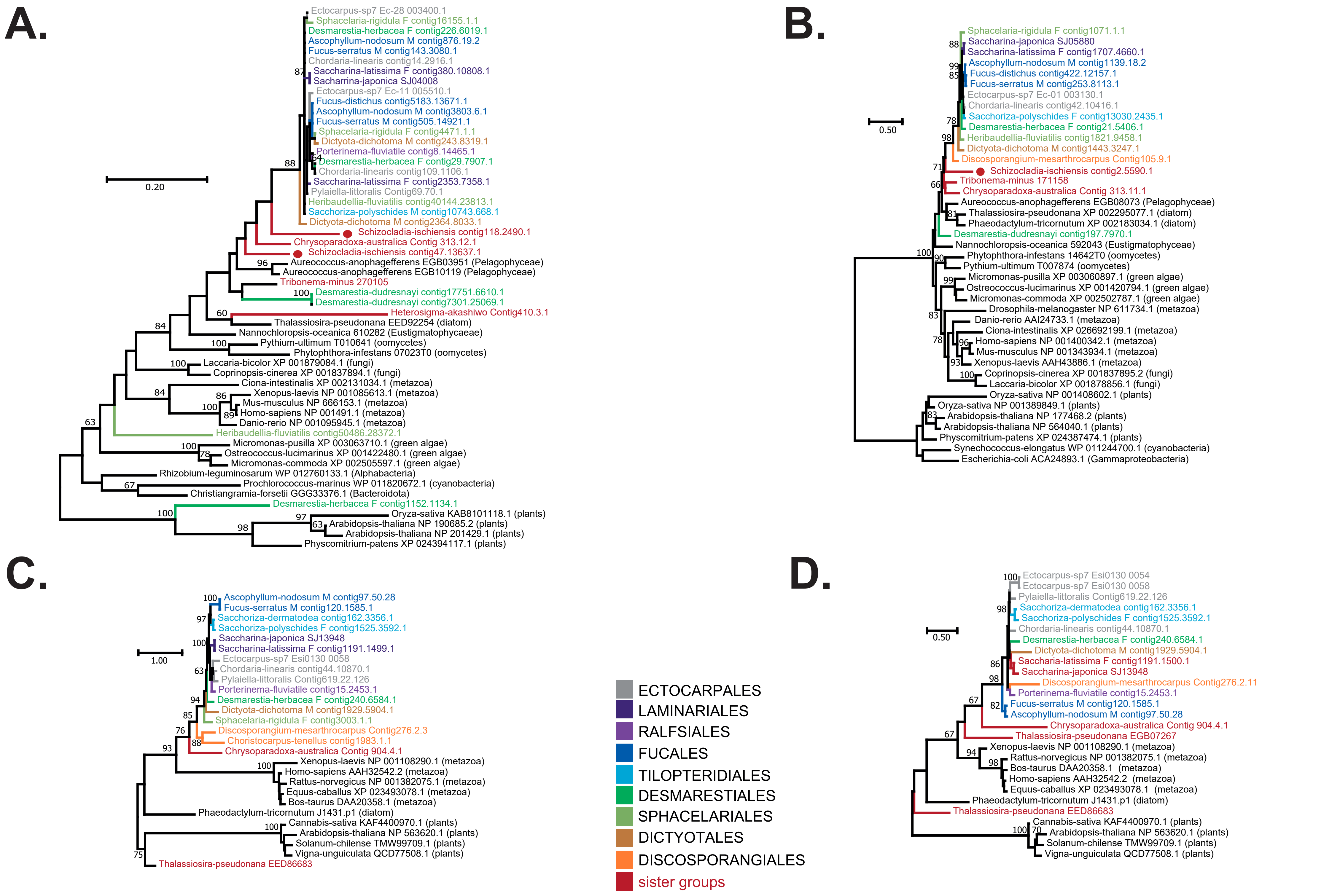

Supplementary Fig. S12

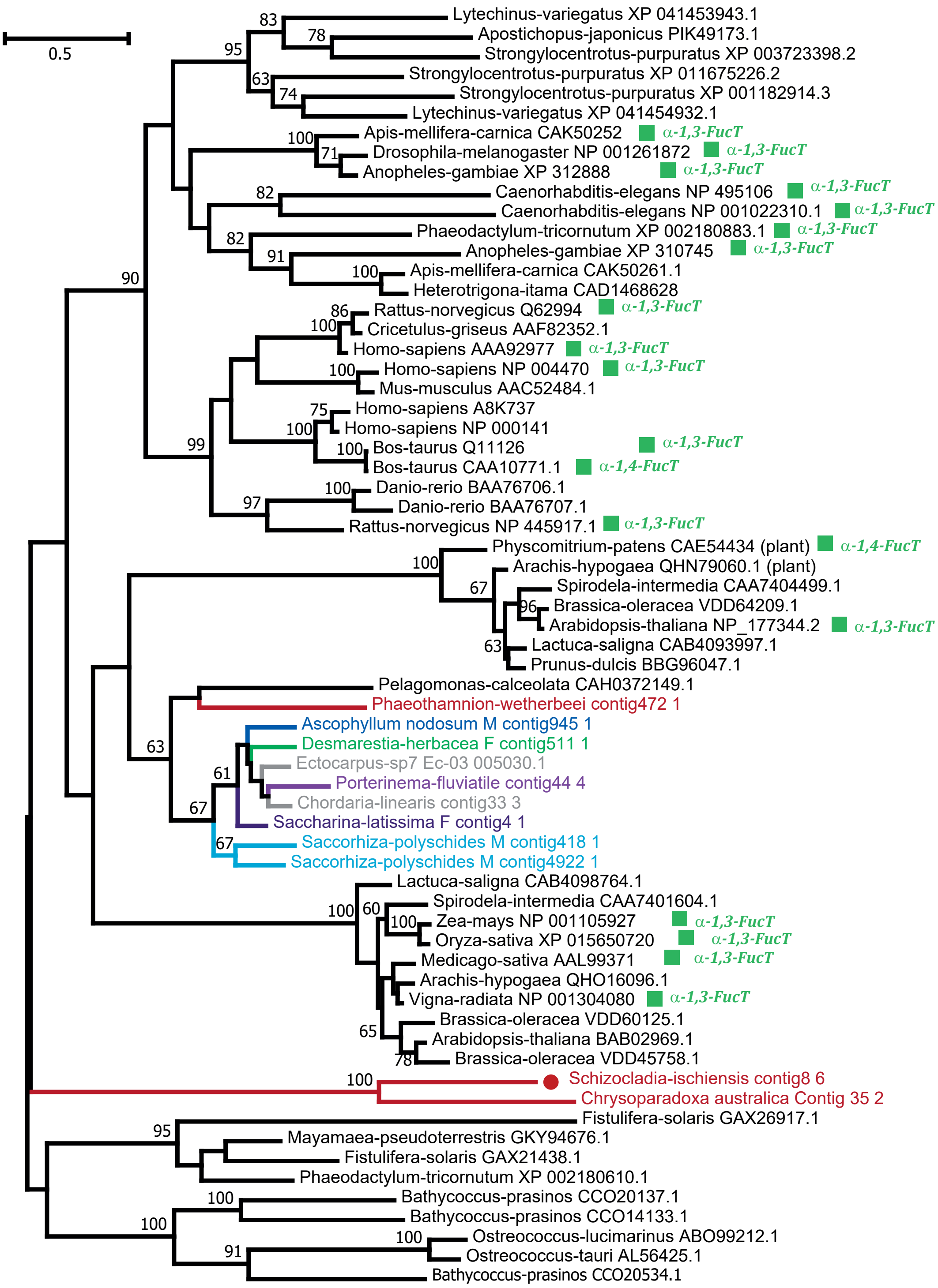

Supplementary Fig. S13

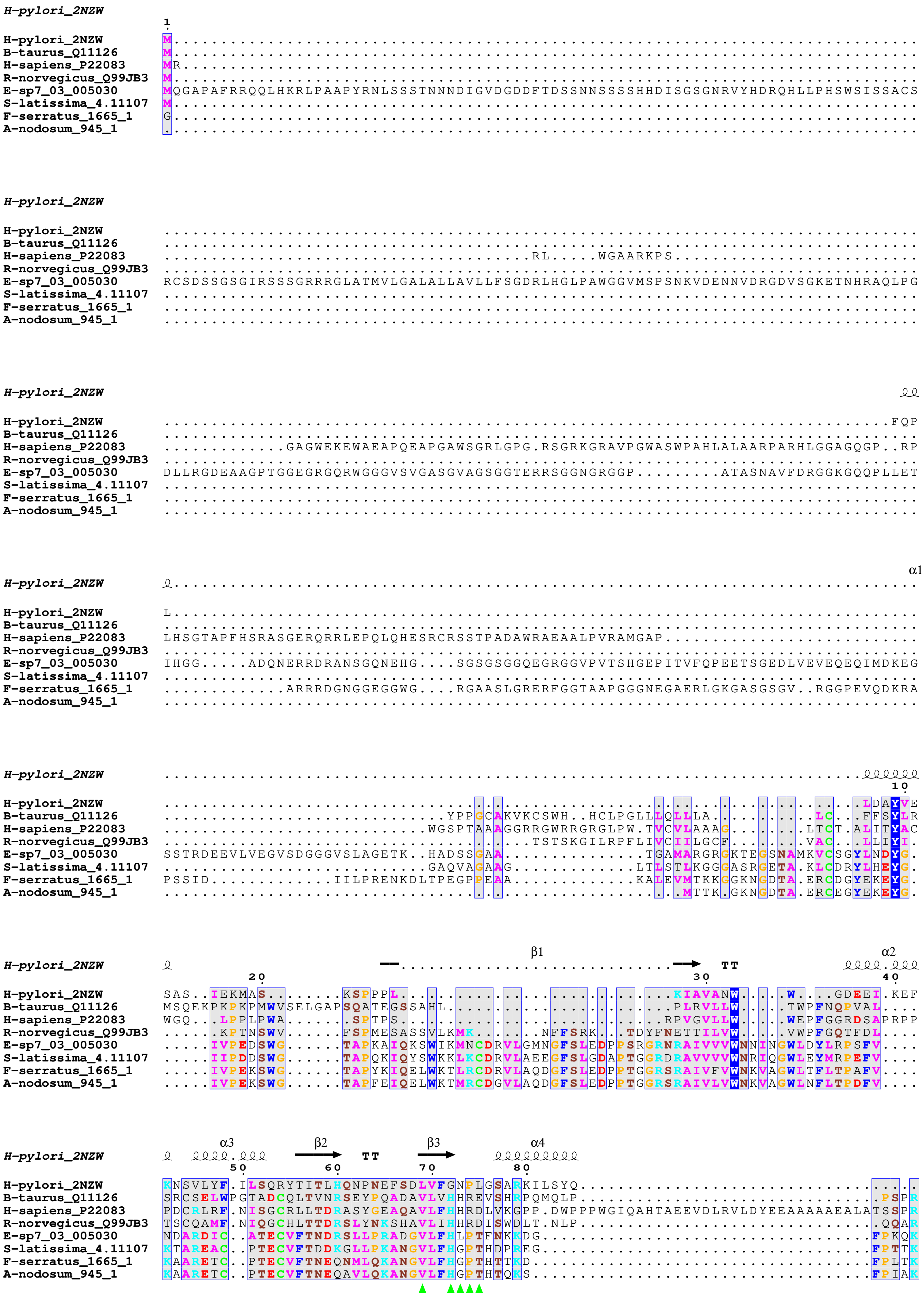
H-pylori_2NZW
B-taurus_Q11126
H-sapiens_P22083
R-norvegicus_Q99JB3
E-sp7_03_005030
S-latissima_4.11107
F-serratus_1665_1
A-nodosum_945_1
1
MQGAPAFRRQQLHKRLPAAPYRNLSSSTNNNDIGVDGDDFTDSSNNSSSSHHDISGSGNRVYHDRQHLLPHSWSISSACS
RL.....WGAARKPS
RCSDSSGSGIRSSSGRRRGLATMVLGALALLAVLLFSGDRLHGLPAWGGVMSPSNKVDENNVDRGDVSGKETNHRAQLPG
FQP
GAGWEKEWAEAPQEAPGAWSGRLGPG.RSGRKGRAVPGWASWPAHLALAARPARHLGGAGQGP..RP
DLLRGDEAAGPTGGEGRGQRWGGGVSVGASGVAGSGGTERRSGGNGRGGP.........ATASNAVFDRGGKGQQPLLET
α1
L
LHSGTAPFHSRASGERQRRLEPQLQHESRCRSSTPADAWRAEAALPVRAMGAP
IHGG....ADQNERRDRANSGQNEHG....SGSGSGGQEGRGGVPVTSHGEPITVFQPEETSGEDLVEVEQEQIMDKEG
ARRRDGNGGEGGWG....RGAASLGRERFGGTAAPGGGNEGAERLGKGASGSGV..RGGPEVQDKRA
10
LDAYVE
YPPGCAKVKCSWH..HCLPGLLLQLLLA
LC...FFSYLR
WGSPTAAAGGRRGWRRGRGLPW.TVCVLAAAG
LTCT.ALITYAC
TSTSKGILRPFLIVCIILGCF
VAC...LLIYI.
SSTRDEEVLVEGVSDGGGVSLAGETK..HADSSGAA
TGAMARGRGKTEGSNAMKVCSGYLNDYG.
GAQVAGAAG
LTLSTLKGGGASRGETA.KLCDRYLHEYG.
PSSID
IILPRENKDLTPEGPPEAA
KALEVMTKKGGKNGDTA.ERCDGYEKEYG.
MTTK.GKNGDTA.ERCEGYEKEYG.
β1
TT
α2
20
30
40
SAS..IEKMAS
KSPPPL
KIAVANW
W
GDEEI.KEF
MSQEKPKPKPMWVSELGAPSQATEGSSAHL
PLRVLLW
TWPFNQPVAL
WGQ...LPPLPWA
SPTPS
RPVGVLLW
WEPFGGRDSAPRPP
KPTNSWV
FSPMESASSVLKMK
NFFSRK
TDYFNETTILVW
VWPFGQTFDL
IVPEDSWG
TAPKAIQKSWIKMNCDRVLGMNGFSLEDPPSRGRNRAIVVVWNNINGWLDYLRPSFV
IIPDDSWG
TAPQKIQYSWKKLKCDRVLAEEGFSLGDAPTGGRDRAVVVVWNRIQGWLEYMRPEFV
IVPEKSWG
TAPYKIQELWKTLRCDRVLAQDGFSLEDPPTGGRSRAIVFVWNKVAGWLTFLTPAFV
IVPEKSWG
TAPFEIQELWKTMRCDGVLAQDGFSLEDPPTGGRSRAIVLVWNKVAGWLNFLTPDFV
α3
β2
TT
β3
α4
50
60
70
80
KNSVLYF.ILSQRYTITLHQNPNEFSDLVFGNPLGSARKILSYQ
SRCSELWPGTADCQLTVNRSEYPQADAVLVHHREVSHRPQMQLP
PSPR
PDCRLRF.NISGCRLLTDRASYGEAQAVLFHHRDLVKGPP.DWPPPWGIQAHTAEEVDLRVLDYEEAAAAAEALATSSPR
TSCQAMF.NIQGCHLTTDRSLYNKSHAVLIHHRDISWDLT.NLP
QQAR
NDARDIC..ATECVFTNDRSLLPRADGVLFHLPTFNKKDG
FPKQK
KTAREAC..PTECVFTDDKGLLPKANGVLFHGPTHDPREG
FPTTK
KAARETC..PTECVFTNEQNMLQKANGVLFHGPTHTTKD
FPLTK
KAARETC..PTECVFTNEQAVLQKANGVLFHGPTHTQKS
FPIAK

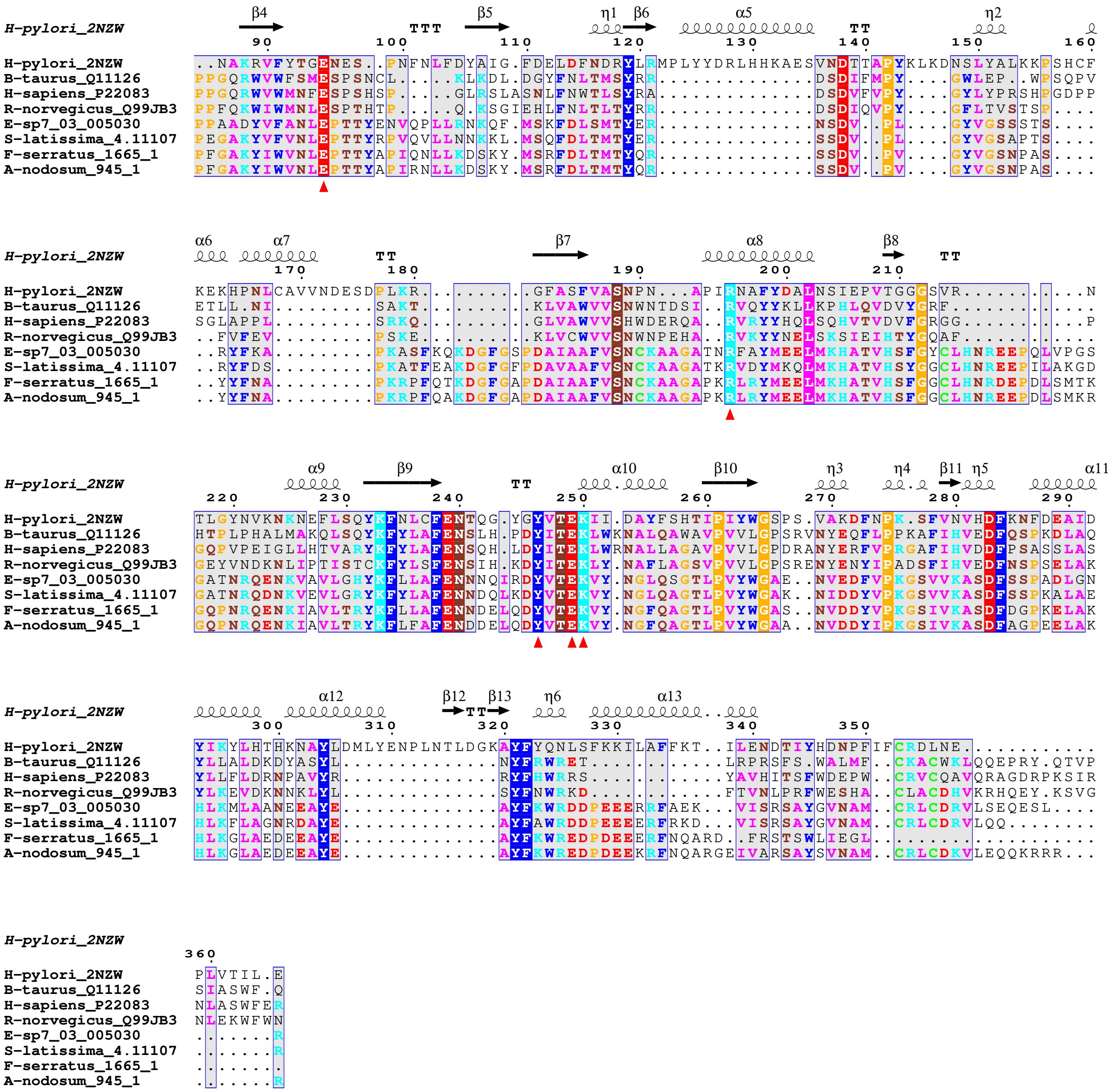

Supplementary Fig. S14

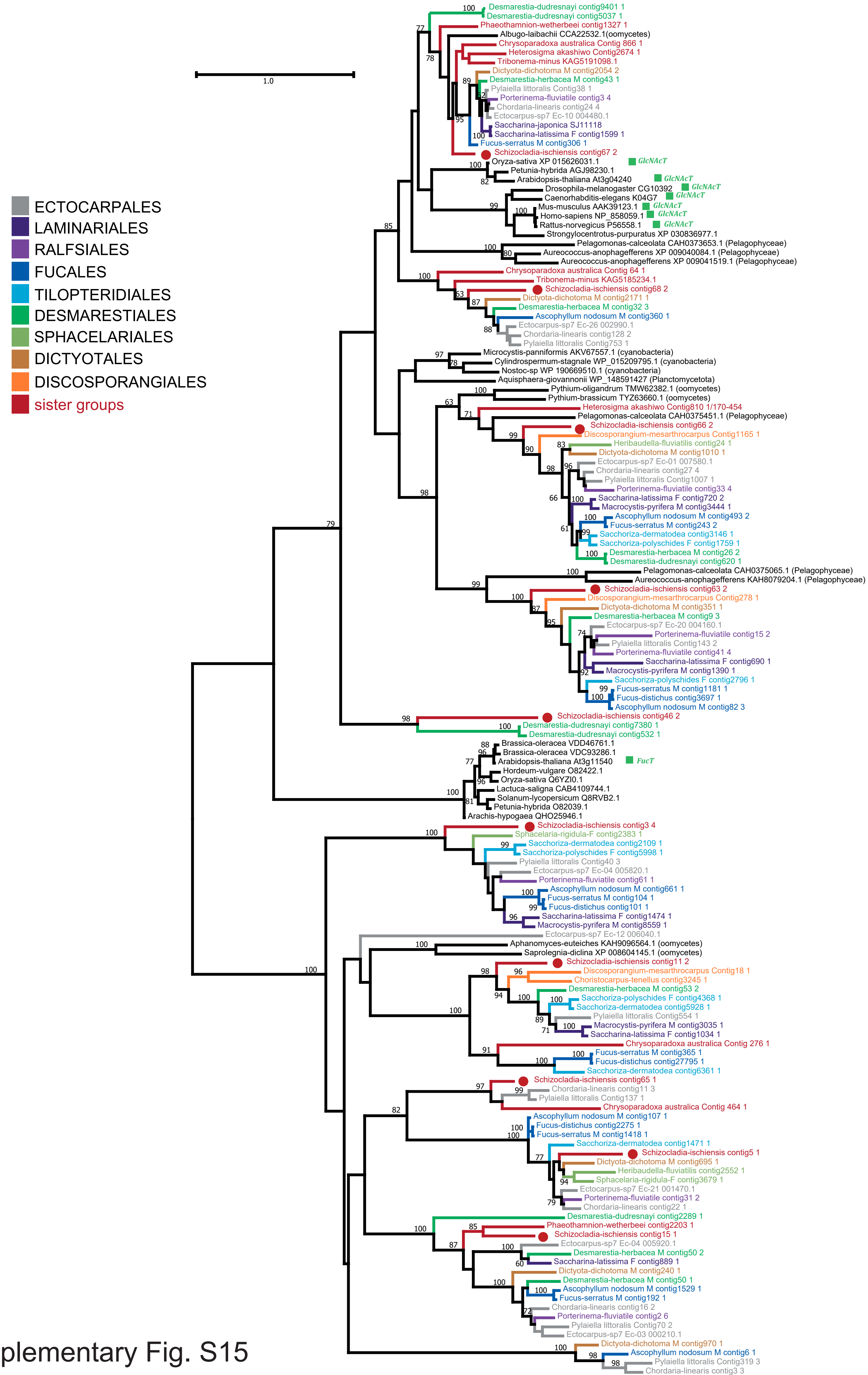

Supplementary Fig. S15

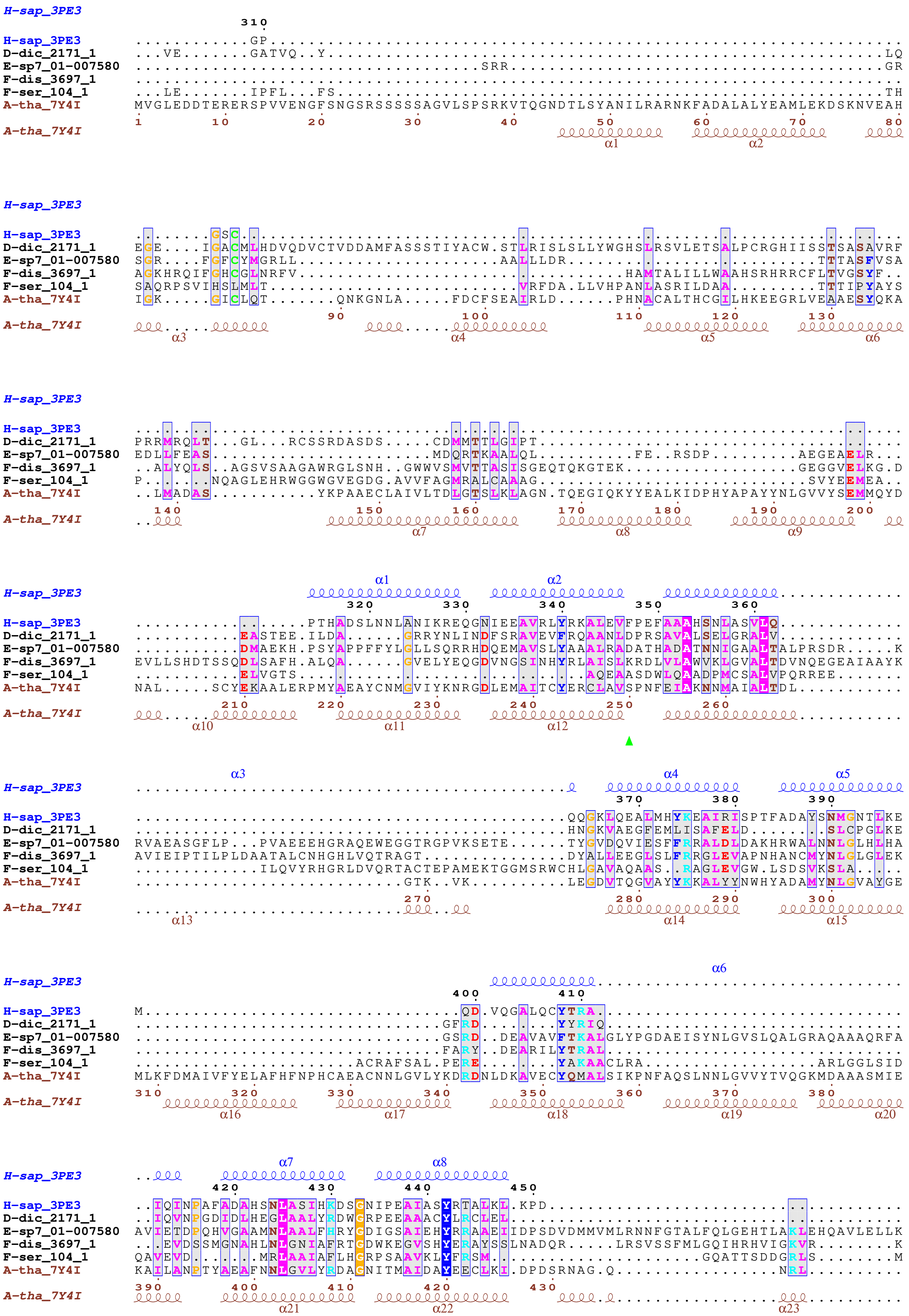
H-sap_3PE3
D-dic_2171_1
E-sp7_01-007580
F-dis_3697_1
F-ser_104_1
A-tha_7Y4I

```
H-sap_3PE3        α9                          α10
                        460       470       480
H-sap_3PE3      ............|.....FPDAYCNLAHCLQIVCDWTDYDERMKKLVSIV.......................................
D-dic_2171_1    ....SAGLPRGSTAVRK.RDDWHVALFKCHAALCDWKGWDTEVDELRAVVQLGRRCEEKESGEDRESGEDPAHAFHGDGE
E-sp7_01-007580 ASDESMPQQRAESGENDVYVDTLVNLHRARKASCDWRDWERDVFDLKYQVD..............................
F-dis_3697_1    AVEY...YKRSYTINND.YSSALASILQSLRSLCLWEDWEVLEPRVASLI...............................
F-ser_104_1     S.......ILGLTMVPP...TIIGSLRTLRQFRVDW.............................................
A-tha_7Y4I      .........LAMNYINEGLDDKLFEAHR......DWG.WR.........................................
                       440       450       460
A-tha_7Y4I                                                                   α24

H-sap_3PE3                                          α11         α12          α13
                                                490       500       510       520
H-sap_3PE3      ...............................ADQLEKNRL.PS.VHPHHSMLYP..LSHGFRKAIAERHGNLCLDKIN.VLH
D-dic_2171_1    SASESVFPDTDGAERSGPKAPKEKHSVRRTSTLERSGIRPV.VHPFDSLSAP..LSASDCLAVAREQSLAVLAESERVLS
E-sp7_01-007580 ...............................VKQLAFGRP.PS.LLPFDTLLLPHPVTPAWRRRLAEAHSARYVEASTALVG
F-dis_3697_1    ...............................MADIDLGRE.PGPQSPYETRLTGE.LKPSYHLGLARLASKRY.DLVTRLPL
F-ser_104_1     ...............................MRDLS.....PMLLSSYRSTAYQGNIRDPY.........GQIGRTPF
A-tha_7Y4I      ...............................FTRLHPQ.YTSWDNLKDPE...............................
                                                     470       480
A-tha_7Y4I                                                     TT

H-sap_3PE3                              η1                                          β1
                530                     540                                        550
H-sap_3PE3      KPPYEHPK..................DLKLS.....DG...............................RLRVGYVSS
D-dic_2171_1    RELPKYTGR..RLADVRWRCRTG..GELMPS.....DG..............................RLRLGYVSG
E-sp7_01-007580 SRSHEAFLGENRTRDAARPEGSDRRTDMQEGIGVEYDGRGEVTGTTAGA....RIHGTTRTDEGRA.....RLKIGFIGH
F-dis_3697_1    KEEHLYTFR..RRLEERAP..........DTSFEVYGGPLKARFQQRGHDFSRKLESHGGEDGTRLVVAYWSF
F-ser_104_1     HMAHQG..............GPD..MELMTALAGVYDAAAPSLRFTAPH..C..RHGVRQDGGKQAQQAWRPTRIGFFSL
A-tha_7Y4I      ...........................................................................RPITIGYISP
                                                                                   490
A-tha_7Y4I                                                                         β1

H-sap_3PE3           α14                                               β2
                   560       570                                      580
H-sap_3PE3      DF.GNHPTSHLMQSIP........GMHNPD..........................KFEVF.....CYA.........LSP
D-dic_2171_1    DLMGSHPLTHLMQSVF........GMHDQG..........................SFEIF.....LYA.........LNP
E-sp7_01-007580 DF.DEHPTAHMIEGVFVWQKRLSGTRQHGTGAADHLTSGAPQKNDWTASMVETNCCRYSVY.....SYGGGGQNAKRTSR
F-dis_3697_1    DL.REHPMGYLTRGLFCS......HQLHNR..........................IFVVTT.SYG.............R
F-ser_104_1     HL.LDHSVGRMMYSLII.......SLAGHP..........................DLEVHVFGPDSYTVGG....ASGP
A-tha_7Y4I      DF.FTHSVSYFIEAPL........THHDYT..........................KYKVV.....VYSA.......VVK
                   500       510                                      520
A-tha_7Y4I          α25                                              β2

H-sap_3PE3         α15      β3  η2      α16           β4          TT   α17      β5
                    590       600       610       620       630       640       650
H-sap_3PE3      DDG.....TNFRVKVMAEANHFIDLSQIPCNGKAADR.IHQDGIHILV.......NMNGYTKGARNE.LFALRPAPIQAM
D-dic_2171_1    ADG.....SRERKRLEHDVEHFRDVSAL.SSLEAAEA.IARDGVSVLV.......NLNGYAGTARSADIFALRPADIAIS
E-sp7_01-007580 GDGGNSTFSRARESIRSCASSFVDLYAD.GHRESVER.IRADGVDILV.......DLQGHTLGGRGQ.ITAARPALVQVN
F-dis_3697_1    NDG.....SFIRDSAEKCAGKFLDVAEL.GDVEAAEV.VARERPEIFV.......DLMAHTTGARPG.IPALKPAPIVAS
F-ser_104_1     SGG.....DHIIDALRSTVHRWHEAPR..NLDTARAVVAQEKLDVLVYPDIGMEALTYFMAFARL........ALVQCV
A-tha_7Y4I      ADAK....TYRFRDKVLKKGGVWKDIYGI.DEKKIASM.VREDKIDILV.......ELTGHTANNKLG.TMACRPAPVQVT
                      530       540       550       560       570       580       590
A-tha_7Y4I          α26       β3  TT      α27          β4       TT TT   α28       β5

H-sap_3PE3               TT   β6 TTT   η3     β7                                     α18
                     660       670       680                                  690
H-sap_3PE3      WLGYPGTSG.ALFMDYIITDQETSPAEVAE.QYSEKLAYM..............................PHTFFIGDHA
D-dic_2171_1    YMGFPGTLGSAELVDYILADRVVIPRELRR.YYAEKVLEM..............................PRCYFVNDYV
E-sp7_01-007580 YLVFPGTSG.APYIDYLLGDRHVTPPEHAR.DFSESLVLL..............................PRTYQANLYE
F-dis_3697_1    YLGYPGTVG.AEFNDYTIADKTVLPPELAEIGFSEKLVYI..............................PDSYQANDYN
F-ser_104_1     WWGHPVTPS.TGAIDYFLS.LDVELQDGQN.DYMEQVVRMDLINTAHFQQACPAKKDAAFFPFPMKKIPCPELFDLRDTE
A-tha_7Y4I      WIGYPNTTG.LPTVDYRITDSLADPPDTKQ.KQVEELVRL..............................PDCFLC....
                      600       610       620                                  630
A-tha_7Y4I               TT   β6 TTT  TT     β7

H-sap_3PE3          η4   β8            β9   α19           β10                  β11      α20
                700       710       720       730       740       750       760       770
H-sap_3PE3      NMFPHLKKKAVIDFKSNGHIYDNRIVLNGIDLKAFLD..SLPDVKIVKMKCPDGGDNADSSNTALNMPVIPMNTIAEAVI
D-dic_2171_1    Q.....SELAVI.........................TSPPVERSGYGLPPGGDDR..........................
E-sp7_01-007580 G....................................LAPT.........................................
F-dis_3697_1    I....................................SHPF..........................................
F-ser_104_1     IP..GYSGSKVGGQRARNHLLEVR.....IPLSYFFSTSLVPKRKVS.......AG...........................
A-tha_7Y4I      .....................................YTPS.........PEAGPVSP.........................
                                                                   640
A-tha_7Y4I                                                         TT
```

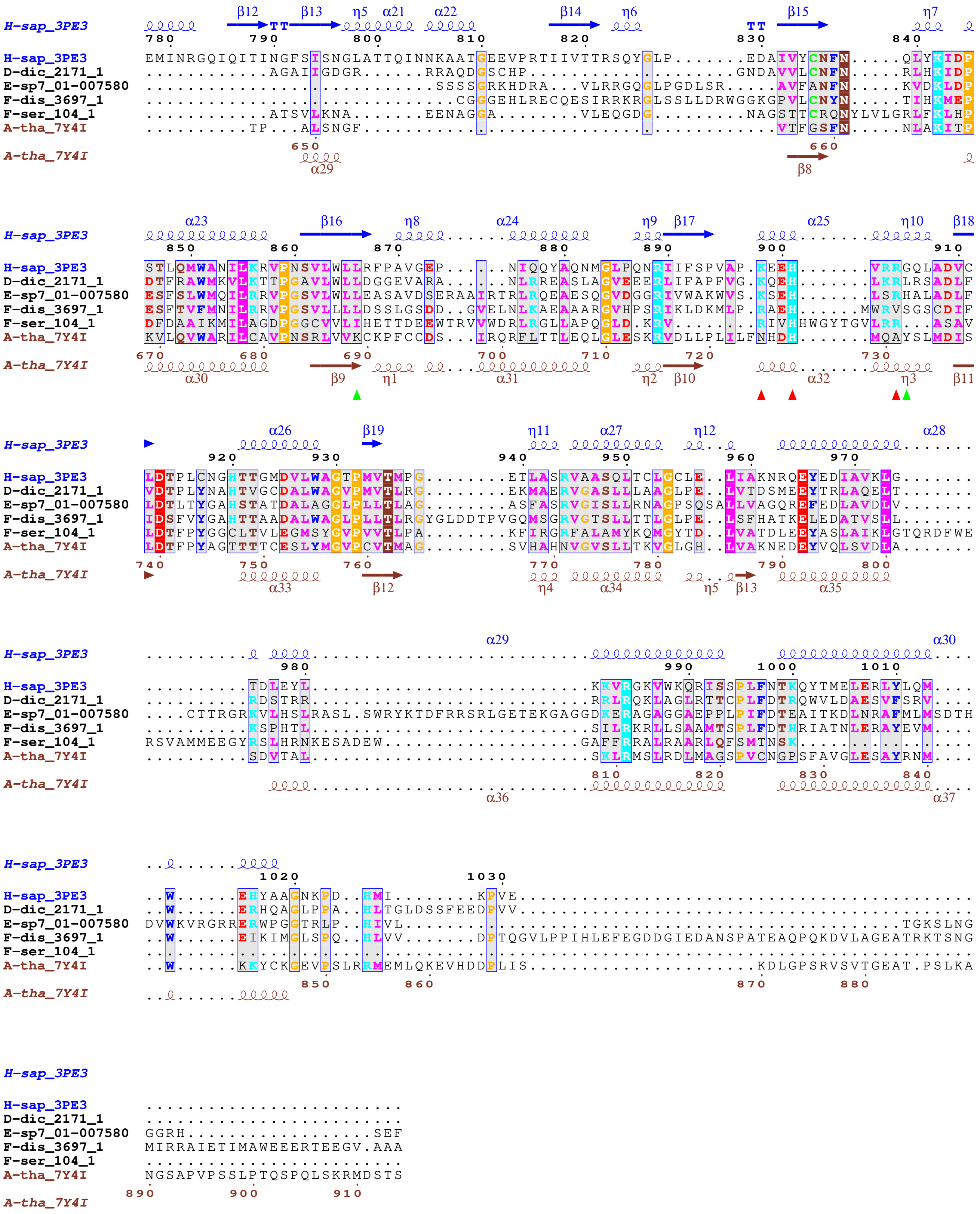

Supplementary Fig. S16

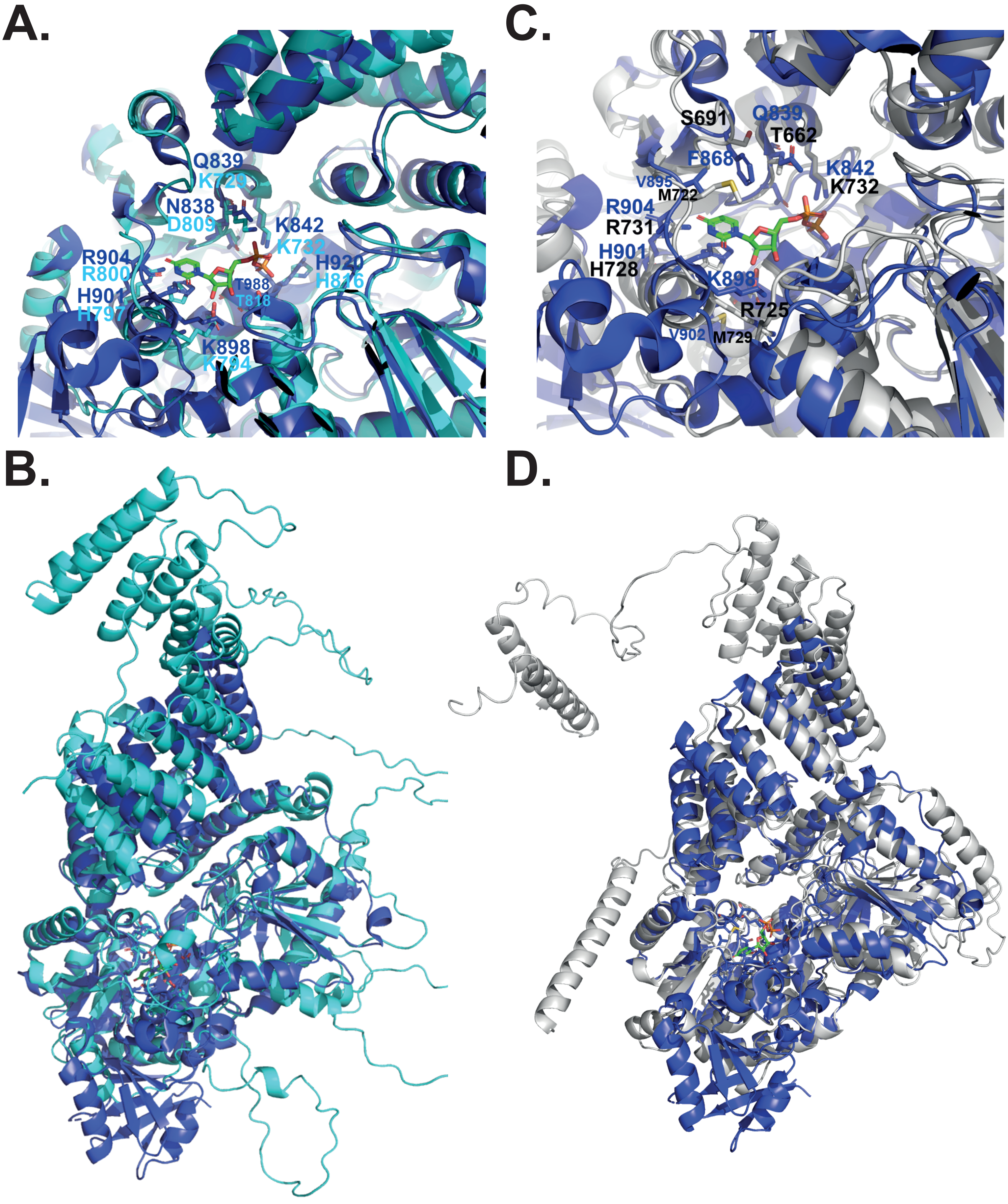

human GT41 (pdb code: 3PE3)
***Ectocarpus* sp7 GT41 (Ec-01_007580_1; AF model)**
***Fucus distichus* GT41 (contig 3697_1; AF model)**

Supplementary Fig. S17

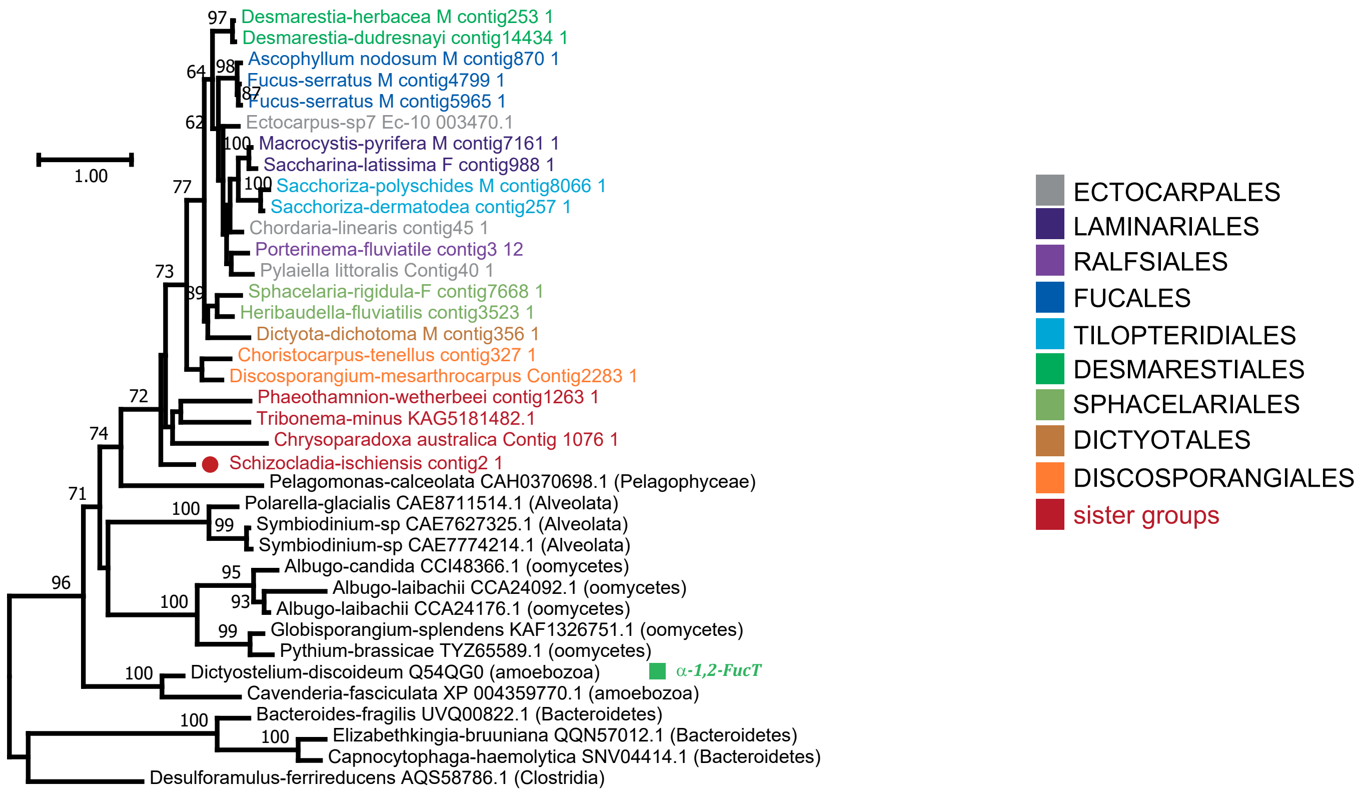

Supplementary Fig. S18

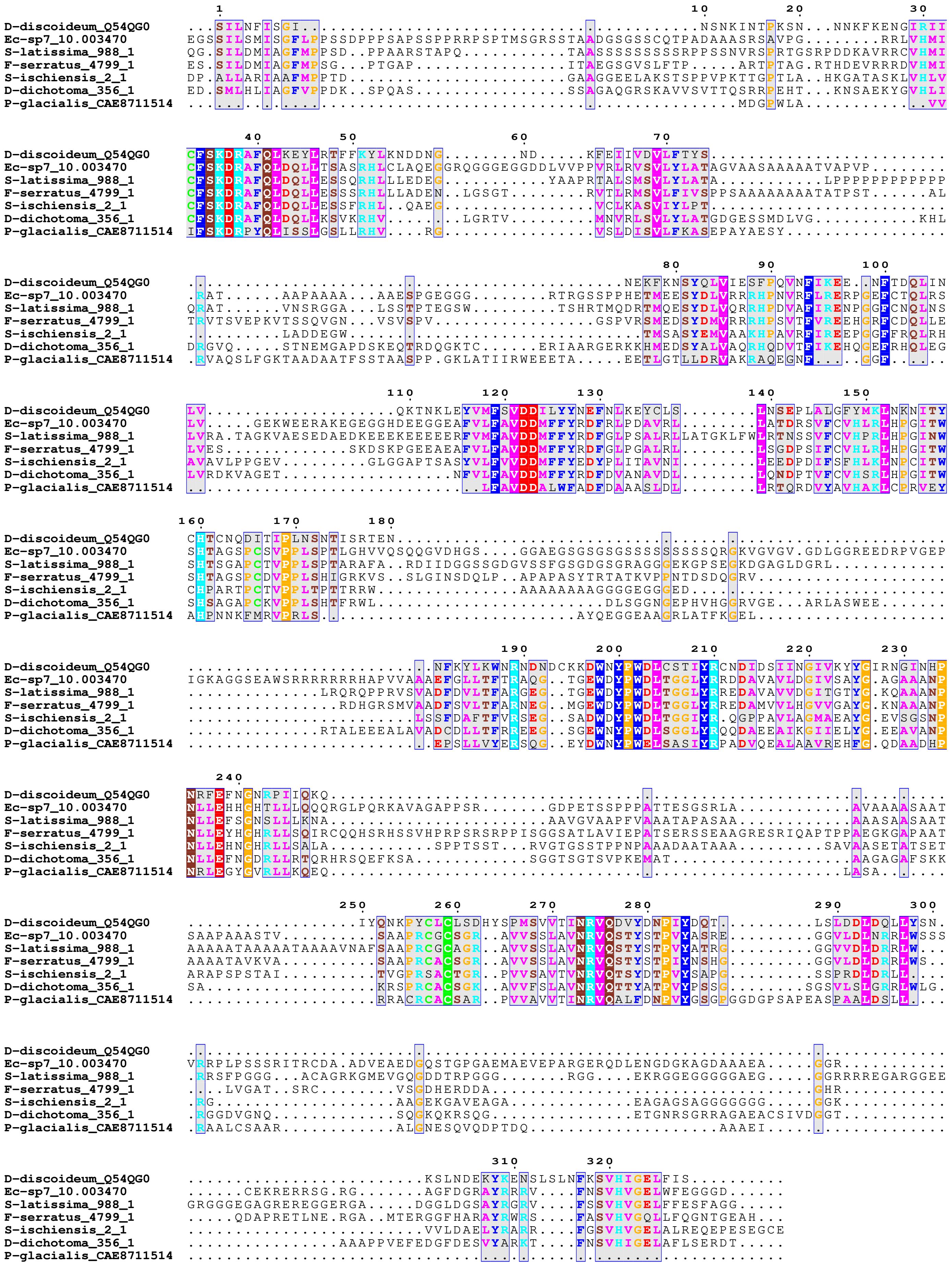

Supplementary Fig. S19

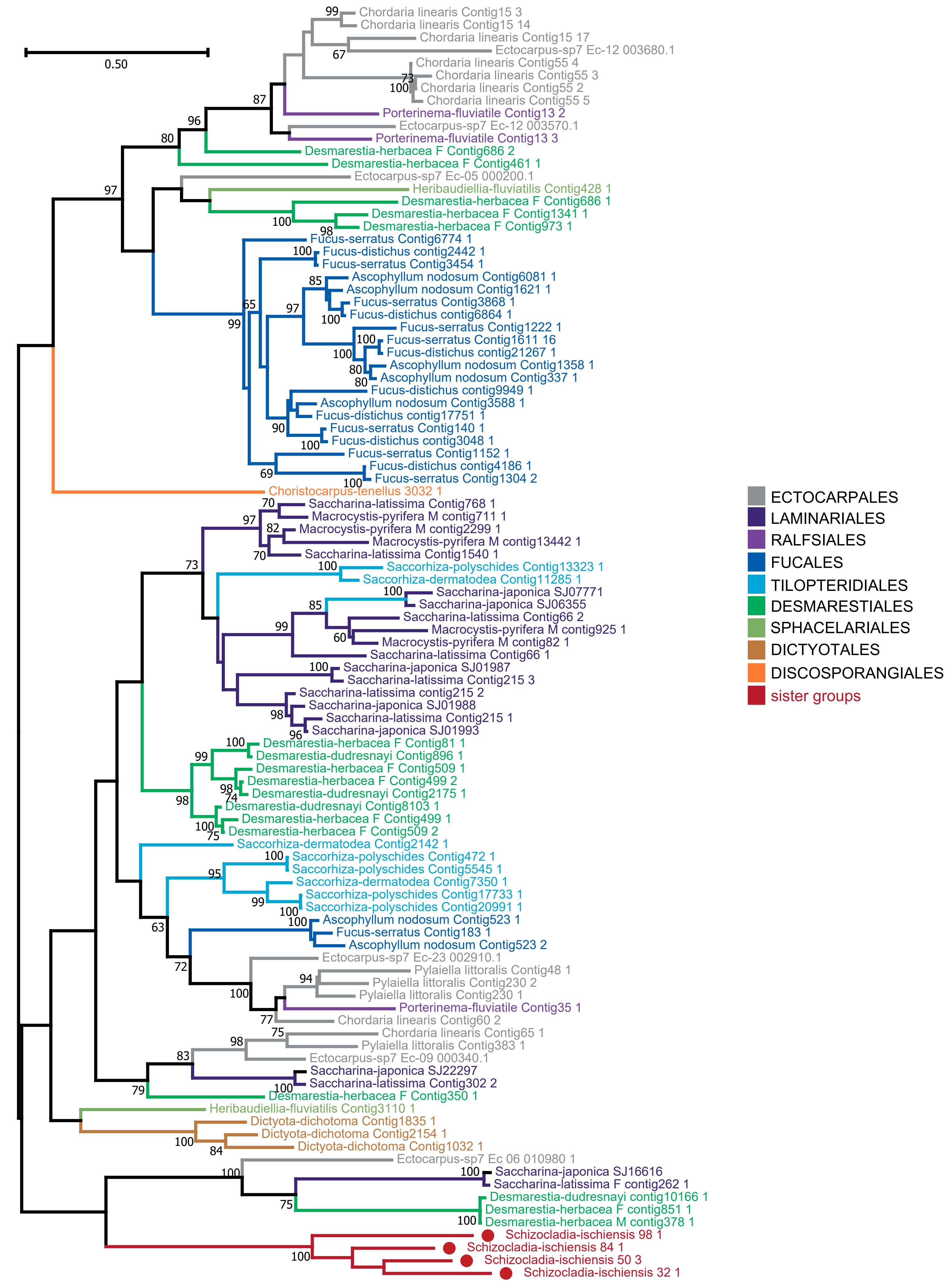

Supplementary Fig. S20

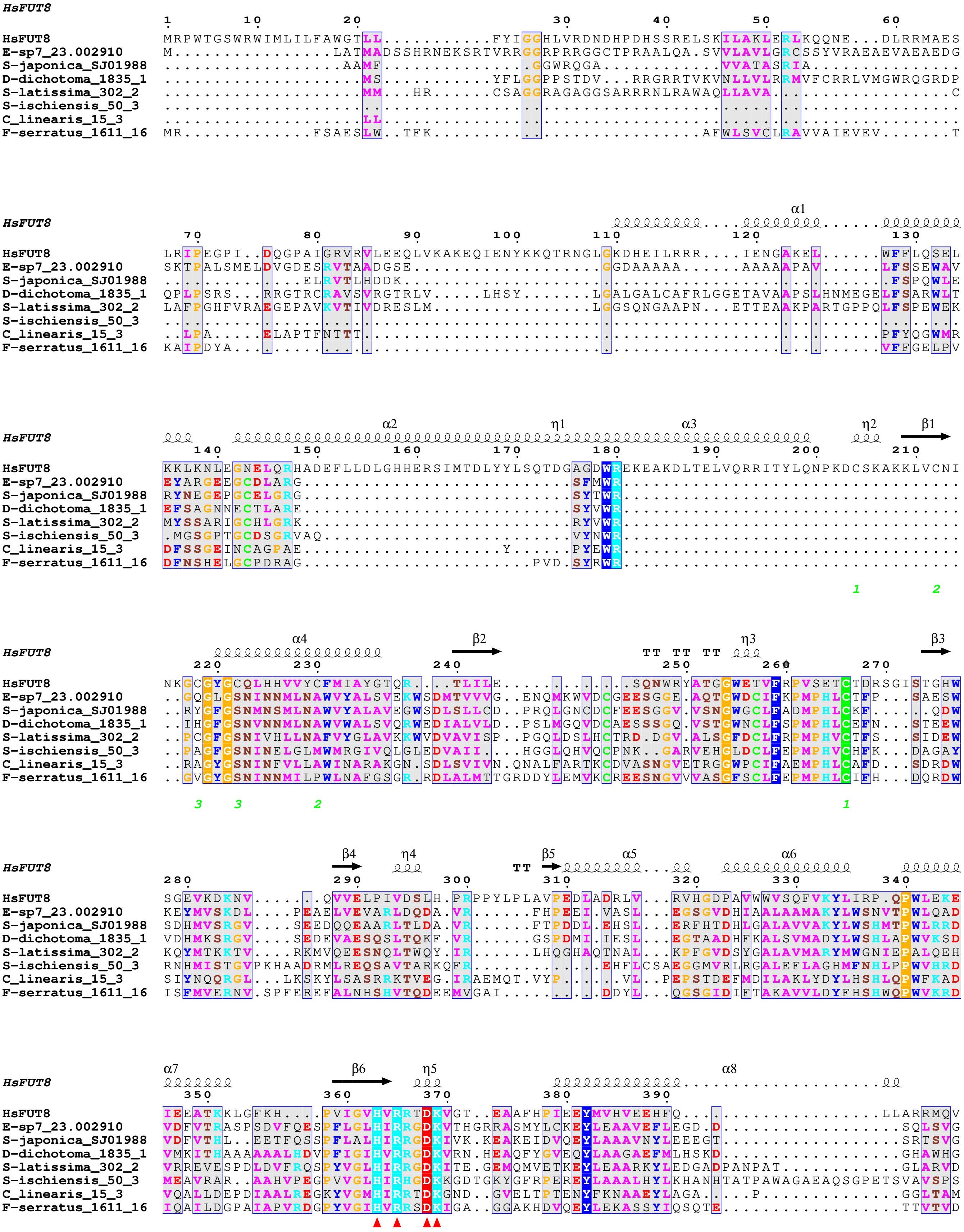
HsFUT8
HsFUT8
E-sp7_23.002910
S-japonica_SJ01988
D-dichotoma_1835_1
S-latissima_302_2
S-ischiensis_50_3
C_linearis_15_3
F-serratus_1611_16
α1
α2
η1
α3
η2
β1
α4
β2
η3
β3
β4
η4
β5
α5
α6
α7
β6
η5
α8

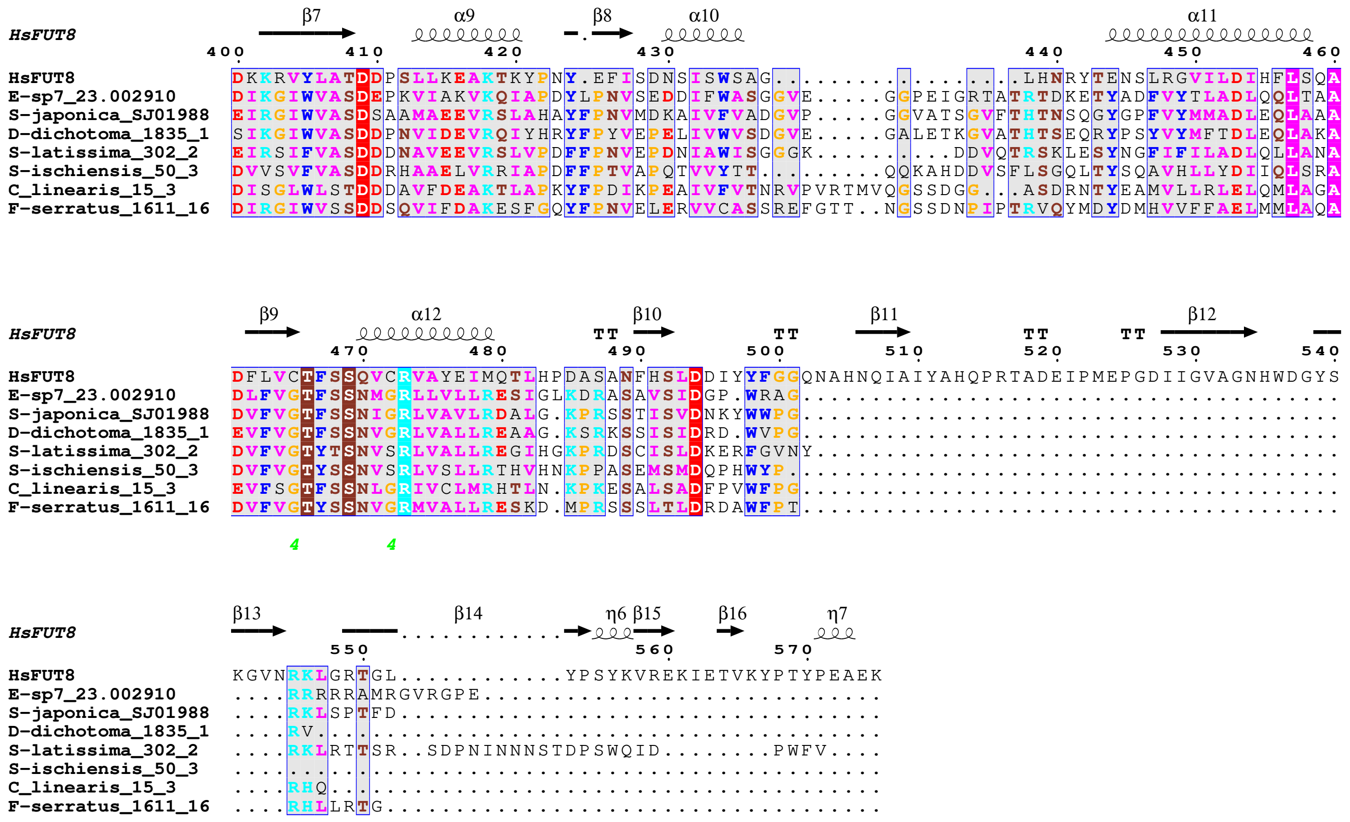

Supplementary Fig. S21

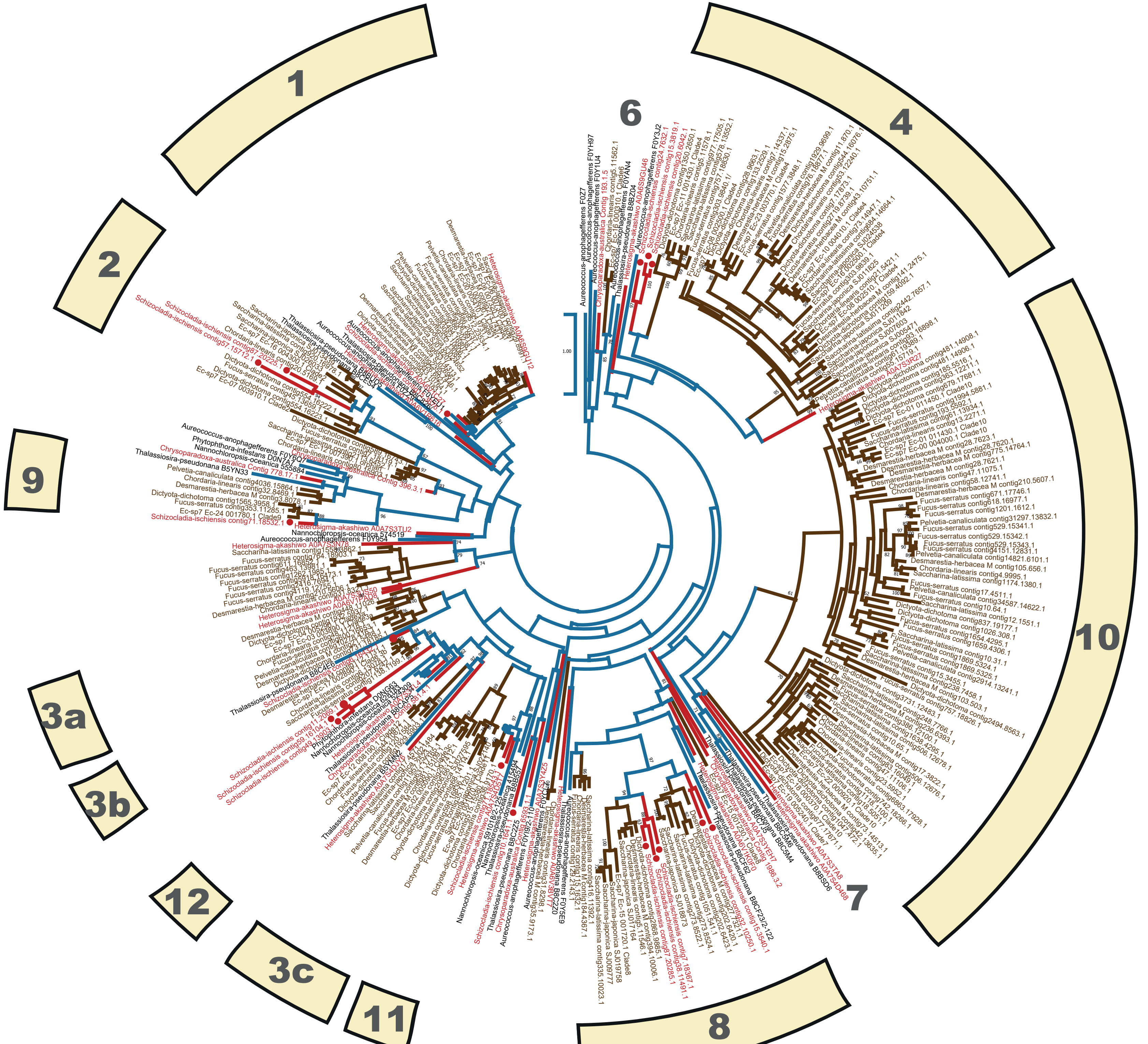

Supplementary Fig. S22

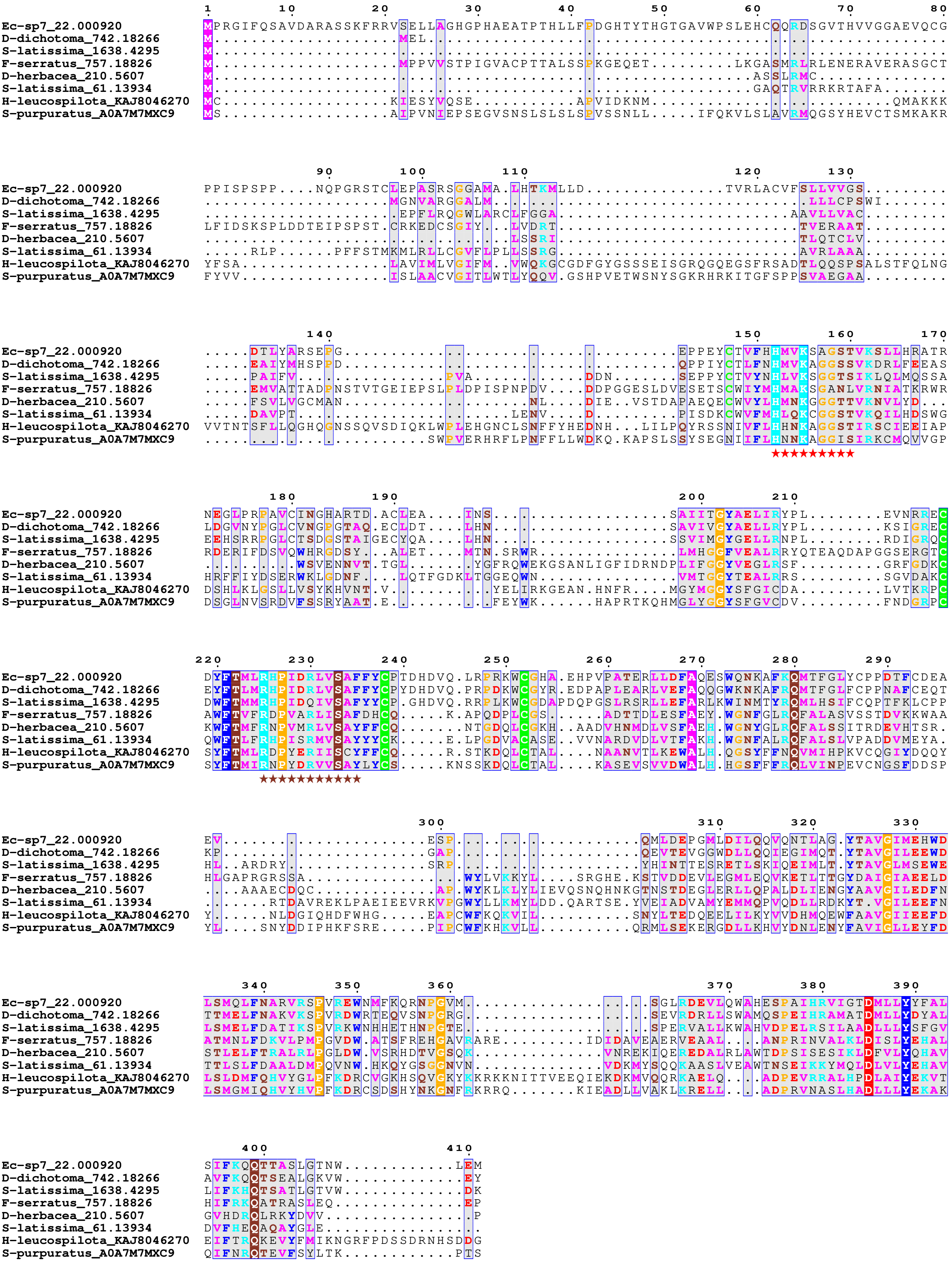

Supplementary Fig. S23
