# Supplementary material for: Candidate genes involved in biosynthesis and degradation of the main extracellular matrix polysaccharides of brown algae and their probable evolutionary history
Source: BMC Genomics. 2024 Oct 10;25:950. doi: 10.1186/s12864-024-10811-3 (PMC11468063; doi:10.1186/s12864-024-10811-3)
Supplement: Supplementary file 2 — Supplementary Material 2 [file 12864_2024_10811_MOESM2_ESM.pdf]

Candidate genes involved in biosynthesis and degradation of the main extracellular matrix polysaccharides of brown algae and their probable evolutionary history

Lisa Mazéas[†,1], Ahlem Bouguerba-Collin[†,1], J. Mark Cock[1], France Denoeud[2], Olivier Godfroy[1], Loraine Brillet-Guéguen[1,3], Tristan Barbeyron[1], Agnieszka P. Lipinska[4], Ludovic Delage[1], Erwan Corre[3], Elodie Drula[5,6,7], Bernard Henrissat[5,6,8], Mirjam Czjzek[1], Nicolas Terrapon[5,6], Cécile Hervé[1,*]

[1]Sorbonne Université, CNRS, Integrative Biology of Marine Models Laboratory, Station Biologique de Roscoff, Roscoff, France

[2]Génomique Métabolique, Genoscope, Institut François Jacob, CEA, CNRS, Université Evry, Université Paris-Saclay, Evry, 91057, France

[3]CNRS, Sorbonne Université, FR2424, ABiMS-IFB, Station Biologique, Roscoff, France

[4]Department of Algal Development and Evolution, Max Planck Institute for Biology, 72076, Tübingen, Germany.

[5]Aix Marseille Univ, CNRS, UMR 7257 AFMB, Marseille, France

[6]INRAE, USC 1408 AFMB, Marseille, France

[7]INRAE, Aix-Marseille Univ., UMR1163 BBF, Marseille, France

[8]Department of Biotechnology and Biomedicine, Technical University of Denmark, Kgs. Lyngby, Denmark

Supplementary Table S1. Gene number of a selection of GT families in brown algae and other relevant organisms and known to contain FucT activities in the CAZy database. All known FucTs are classified in the CAZy GT families as shown. The generated glycosidic linkages identified from characterized enzymes (outside brown algae) are indicated. The average value is shown for the following species: *Ectocarpus* sp.7, *Pylaiella littoralis, Chordaria linearis* (Ectocarpales), *Saccharina latissima, Saccharina japonica, Macrocystis pyrifera* (Laminariales), *Porterinema fluviatiles* (Ralfsiales), *Ascophyllum nodosum, Fucus serratus, Fucus distichus* (Fucales), *Saccorhiza dermatodea, Saccorhiza polyschides* (Tilopteridiales), *Desmarestia herbacea*, *Desmarestia dudresnayi* (Desmarestiales), *Sphacelaria rigidula* (Sphacelariales), *Dictyota dichotoma* (Dictyotales), *Discosporangium mesarthrocarpum, Choristocarpus tenellus* (Discosporangiales), *Schizocladia ischiensis* (Schizocladiophyceae), *Heterosigma akashiwo* (Raphidophyceae), *Phaeodactylum tricornutum CCAP 1055, Thalassiosira pseudonana* CCMP 1335*, Chaetoceros tenuissimus* NIES-3715_426638 (diatoms), *Lytechinus variegatus* NC3_7654, *Strongylocentrotus purpuratus* Spur-01_7668 (sea urchin), *Apostichopus japon* Shaxun_307972 (sea cucumber). ND. Not determined.

**GT families**	**GT10**	**GT23**	**GT41**	**GT74**	**GT11**	**GT37**	**GT65**	**GT68**
Known FucT activities in CAZy	α-1,3/4-FucTs	α-1,6-FucTs	protein O-FucTs	α-1,2-FucTs	α-1,2/3-FucTs	α-1,2-FucTs	protein O-FucTs	protein O-FucTs
**Phaeophyceae**								
Ectocarpales [3]	**1**	**9**	**12**	**1**	0	0	0	0
Laminariales [3]	**1**	**9**	**9**	**1**	0	0	0	0
Ralfsiales [1]	**3**	**7**	**11**	**1**	0	0	0	0
Fucales [3]	**1**	**14**	**10**	**1**	0	0	0	0
Tilopteridiales [2]	**0**	**5**	**10**	**2**	0	1	0	0
Desmarestiales [2]	**1**	**11**	**15**	**1**	0	1	0	0
Sphacelariales [1]	**0**	**1**	**6**	**1**	0	0	0	0
Dictyotales [1]	**1**	**4**	**9**	**1**	0	1	0	0
Discosporangiales [2]	**1**	**1**	**5**	**1**	0	1	0	0
**Sister groups to Phaeophyceae**								
Schizocladiophyceae [1]	**1**	**6**	**11**	**1**	0	1	0	0
Raphidophyceae [1]	**0**	**0**	**8**	**0**	0	0	0	0
**Other Stramenopiles**								
diatoms [3]	**3**	**0**	**2**	**1**	ND	ND	ND	ND
**Echinoderms**								
Sea urchin [2]	**60**	**7**	**4**	**0**	ND	ND	ND	ND
Sea cucumber [1]	**97**	**1**	**1**	**0**	ND	ND	ND	ND
